# Perfluoroalkyl Substances in Seabird Eggs from Canada’s Pacific Coast: Temporal Trends (1973–2019) and Interspecific Patterns

**DOI:** 10.1021/acs.est.3c02965

**Published:** 2023-07-13

**Authors:** Robert Kesic, John E. Elliott, Kyle H. Elliott, Sandi L. Lee, France Maisonneuve

**Affiliations:** † Wildlife Research Division, Environment and Climate Change Canada, Delta V4K 3N2, British Columbia, Canada; ‡ Department of Natural Resource Sciences, McGill University, Sainte Anne-de-Bellevue H3A 0G4, Quebec, Canada; § Environment and Climate Change Canada, National Wildlife Research Centre, Ottawa K1A 0H3, Ontario, Canada

**Keywords:** seabird eggs, PFOS, PFASs, long-chain PFCAs, temporal trends

## Abstract

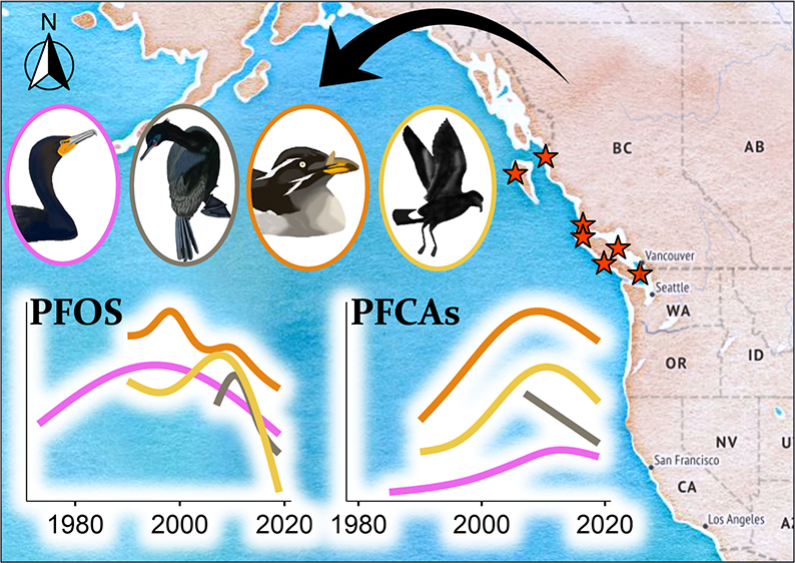

Whether perfluoroalkyl sulfonates (PFSAs) and perfluoroalkyl carboxylates (PFCAs) are responding to legislative restrictions and showing decreasing trends in top marine predators that range across the eastern North Pacific Ocean is unclear. Here, we examined longer-term temporal trends (1973–2019) of 4 PFSAs and 13 PFCAs, as well stable isotopes of δ^13^C and δ^15^N, in the eggs of 4 seabird species sampled along a nearshore-offshore gradient; double-crested cormorants (*Nannopterum auritum*), pelagic cormorants (*Urile pelagicus*), rhinoceros auklets (*Cerorhinca monocerata*), and Leach’s storm-petrels (*Hydrobates leucorhous*) from the Pacific coast of British Columbia, Canada. PFOS was the most abundant PFSA (79–94%) detected in all eggs regardless of colony and year, with the highest concentrations, on average, measured in auklet eggs (mean = 58 ng g^–1^, range = 11–286 ng g^–1^ ww). Perfluoroundecanoic acid (PFUdA) and perfluorotridecanoic acid (PFTriDA) were the dominant long-chain PFCAs (≥30% combined). The majority of PFSAs (including PFOS) are statistically declining (*p* < 0.001) in the eggs of all 4 species with PFOS half-lives ranging from 2.6 to 7.8 years. Concentrations of long-chain PFCAs exhibited a trajectory comprised of linear increases and second-order declines, suggesting that the rate of uptake of PFCAs is slowing or leveling off. These trends are consistent with the voluntarily ceased production of PFSAs by 3M circa 2000–2003 and are among the first from the northeast Pacific to indicate a positive response to several regulations and restrictions on PFCAs from facility emissions and product content.

## Abstract 2

Much less is known about temporal trends of PFASs in top predators that range across the North Pacific Ocean. This study reports decreasing trends in PFAS concentrations in the eggs of 4 seabird species sampled along a nearshore-offshore gradient.

## Introduction

Poly- and per-fluoroalkyl substances (PFASs) are a group of synthetic organofluorine compounds, exhibiting both hydrophobic and hydrophilic properties.^1^ PFASs are generally classified into two groups: (1) perfluoroalkyl acids, which include the perfluoroalkyl sulfonates (PFSAs) and perfluoroalkyl carboxylates (PFCAs); and (2) polyfluoroalkyl substances, which includes the fluorotelomer alcohols (FTOHs), monomers, olefins, iodides, and ether acids,^2^ some of which are transformed abiotically/biotically into perfluoroalkyl substances.^3^ Due to the strong and stable C–F bond imparted by the perfluoroalkyl moiety, PFASs and their salts have been used in various industrial processes and consumer products since the 1950s, including surface coatings of apparel, industrial and home furnishings, paper protection (e.g., food packaging), and performance use (e.g., surfactants, hydraulic fuel additives, pharmaceuticals, insecticides, cleaners, and Class B firefighting aqueous film-forming foams, AFFFs).^1,4^

PFASs have been recognized as global contaminants of concern due to their persistence, bioaccumulation, toxicity, and long-range transport (LRT) properties.^3,5−7^ In 2000, 3M announced the phase-out of perfluorooctane sulfonyl fluoride (POSF)-based products, including perfluorooctane sulfonic acid (PFOS), followed by the actual phase-out in 2003.^8^ In 2009, PFOS, its salts, and precursors were listed under Annex B (restrict production and use) in the Stockholm Convention on Persistent Organic Pollutants (POPs). That eventually led to the collaborative and voluntary elimination of perfluorooctanoic acid (PFOA) in 2019, and more recently, perfluorohexane sulfonate (PFHxS) and its products under Annex A in the Convention (www.pops.int). In 2006, Environment and Climate Change Canada (ECCC) and Health Canada embarked on an Environmental Performance Agreement with four companies to reduce PFOA, and other long-chain PFCAs in perfluorinated chemicals in Canadian commerce by 95% no later than 2010, and to eventually eliminate the remaining 5% by 2015.^19^ In addition, eight leading fluoropolymer and fluorotelomer manufacturers joined the US Environmental Protection Agency (US EPA) PFOA Stewardship Program with the goal of reducing (and eliminating) PFOA/PFCAs from facility emissions and product content by the end of 2015.^3,38^

Despite efforts to limit the production, use, and distribution of PFASs in North America, historic emissions are still in circulation. Since 2003, China has voluntarily increased production of PFOS-based products to up to ∼200 tons per year to accommodate domestic demands and overseas needs.^9^ The production and usage of short- and long-chain PFCAs (defined as C_*n*_F_2*n*+1_COOH, *n* ≥ 7)^10^ and their precursors in China, India, and Russia have also increased following phase-outs in North America, with up to ∼22,000 tons of C_4_-C_14_ PFCAs emitted globally from 1951 to 2012 (based on the life-cycle of fluorotelomer-based products) and up to ∼6500 tons to be emitted from 2016 to 2030 based on commitments to the US EPA PFOA Stewardship Program.^6^ Consequently, there is ongoing concern that exposure to PFASs could vary spatially and temporally at local, regional, and global scales, particularly in marine environments which act as final “sinks” for terminal PFASs and other contaminants.^11,12^

Seabirds are effective sentinels for monitoring contaminants in marine systems because they are long-lived, widely distributed, and feed at relatively high trophic levels,^13^ suitably integrating contaminant exposure across space and time.^14,16^ Seabird eggs are a particularly useful sampling matrix for monitoring PFASs and other contaminants because they are (1) easy to identify and collect; (2) relatively noninvasive; (3) easily homogenized; (4) generally representative of the contaminant burden in the female adult prior to and during egg-laying; (5) collected from multiple breeding colonies, facilitating a large sample size and high statistical power; and (6) a rich source of lipids and proteins where contaminants are mobilized and partitioned.^15,16^ Legacy and emerging contaminants have been monitored in seabird eggs from the Pacific coast of British Columbia (BC), Canada, since 1968.^16−19^ Eggs have been sampled from four seabird species along a nearshore-offshore gradient: double-crested cormorants (*Nannopterum auritum*; DCCO), pelagic cormorants (*Urile pelagicus*; PECO), rhinoceros auklets (*Cerorhinca monocerata*; hereafter auklet; RHAU), and Leach’s storm-petrels (*Hydrobates leucorhous*; hereafter storm-petrel; LSPE). These four seabird species have different foraging habitats and diets,^16,18,56,57^ and therefore, measuring contaminant levels in their eggs provides key information regarding the temporal and spatial variation of PFASs and other contaminants along the BC coast, and more broadly, the eastern North Pacific Ocean.

Between 1973 and 2011, overall decreasing trends were seen for PFOS in the eggs of double-crested cormorants and auklets (but not storm petrels) sampled from various colonies along the BC coast.^19^ Conversely, increasing trends were seen for perfluoroundecanoic acid (PFUdA) and perfluorotridecanoic acid (PFTriDA) in the eggs of auklets and storm petrels, while those in cormorant eggs showed no major trends.^19^ However, temporal trends for other long-chain PFCAs were not transparent in the aforementioned study due to insufficient sample sizes and/or low detection frequencies. Hence, there is a need to re-evaluate the bioaccumulation and biomagnification potential of PFASs in these seabird food webs to determine whether PFAS levels are responding to changes in regulations and/or dietary tracers, such as stable isotopes of carbon (δ^13^C) and nitrogen (δ^15^N) over time. Notably, the eastern North Pacific Ocean warrants significant attention as it remains an understudied region in coastal North America and is often subject to LRT (via oceanic and atmospheric currents) of PFASs and other contaminants from various Asian and North American sources.^4,12^

The objectives of the present study were therefore to (1) examine longer-term temporal trends (1973–2019) of 4 PFSAs and 13 PFCAs in the eggs of four seabird species sampled along a nearshore-offshore gradient off the Pacific coast of BC, Canada; (2) investigate whether voluntary phase-outs and international restrictions on PFSAs and PFCAs has had a noticeable effect in the eggs of our monitoring species; (3) compare egg concentrations of PFASs among coastal and offshore species; and (4) evaluate interspecific patterns in relation to habitat use and dietary uptake of PFASs via stable isotope analyses of δ^15^N and δ^13^C in eggs.

## Materials and Methods

### Species Sampling Sites and Egg Collections

Seabird eggs were generally collected every 3–4 years (as part of the long-term contaminants monitoring program initiated in 1968 by the Canadian Wildlife Service^17^) from various colonies located along the Pacific coast of BC, Canada (Figure S1). The selection of seabirds as bioindicators of contamination is predicated on the niche partitioning of species by habitat and diet, their individual ecological characteristics, and the oceanography of the region to be monitored. Double-crested cormorants (1973–2019) were selected due to their year-round residency and short-distance migratory behavior along the coast, their nearshore benthic feeding habits (schooling fish, invertebrates), and their high sensitivity to organic contaminants.^18,25^ Routine monitoring began a few years later and eventually expanded to other year-round/short-range species, such as pelagic cormorants (2007–2019) breeding on Mitlenatch Island, permitting better spatial coverage of the Salish Sea.^16^ The continental shelf and offshore/pelagic zones were covered by auklets (1990–2019) and storm petrels (1990–2019), respectively, with both of these species typically preying on zooplankton, small fish, and/or other juvenile prey.^16,18^ Although pelagic cormorants were not monitored until the early 2000s, their egg collections span roughly the same amount of time as the other three species during that decade and still provide sufficient data to detect trends in cormorant populations from the Canadian Pacific coast.

Sampling sites were selected based on size, history, and accessibility. To standardize egg collections, a single freshly laid/unincubated egg was collected from an active nest early in the breeding season. Internal contents were transferred to chemically rinsed (acetone/hexane) jars and stored frozen (−40 °C) at the National Wildlife Research Centre and the National Wildlife Specimen Bank in Ottawa prior to analyses. Each freezer was monitored for ideal temperatures and protected from electrical failure to maintain sample integrity. Due to cost and other logistics, eggs in the 1990s were analyzed as superpools (5–7 pools of 3 eggs). In all other years, eggs were analyzed as 5 pools of 3 eggs per species. Eggs were collected under research permits authorized by ECCC. Details about species, sites, and egg collections have been described previously.^16,19^

### Chemical Analyses

The full list of Σ_4_PFSAs analyzed included PFOS, perfluorobutane sulfonic acid (PFBS), PFHxS, and perfluorodecane sulfonic acid (PFDS). The full list of Σ_13_PFCAs analyzed included perfluorobutanoic acid (PFBA), perfluoropentanoic acid (PFPeA), perfluorohexanoic acid (PFHxA), perfluoroheptanoic acid (PFHpA), PFOA, perfluorononanoic acid (PFNA), perfluorodecanoic acid (PFDA), PFUdA, perfluorododecanoic acid (PFDoA), PFTriDA, perfluorotetradecanoic acid (PFTeDA), perfluorohexadecanoic acid (PFHxDA), and perfluorooctadecanoic acid (PFODA). Σ_17_PFASs = Σ_4_PFSAs + Σ_13_PFCAs.

Approximately 1.0 g of each egg pool sample homogenate was accurately weighed, transferred into a 15 mL PE centrifuge tube, spiked with IS solution, and 10 mL of 1% acetic acid in acetonitrile was added. A 1.25 g aliquot of a 4:1 magnesium sulfate/sodium acetate salt mixture was added to the 10 mL homogenate before the tube was vortexed and centrifuged. The resulting supernatant was transferred into a UCT QuEChERS tube (Chromatographic Specialties ECQUUS1215CT) to which 50 mg of SupelClean ENVI-18 was previously added. The extract was subsequently shaken and centrifuged again. The final supernatant was transferred and evaporated to dryness before being reconstituted in 1 mL of water/methanol 25:75. The final extracts were filtered through a 0.22 μm Nylon centrifuge filter and transferred into a 300 μL PFC-free polypropylene plastic autosampler vial prior to injection. Compounds in egg samples were quantified using an Agilent 1260/90 HPLC system equipped with a trap column (X-Terra MS; 3.5 μm, 3 × 100 mm; WATERS 186000412) and coupled to a triple quadrupole mass spectrometer (Sciex API 5500) with the TurboSpray ion source in negative polarity using Scheduled Multiple Reaction Monitoring. An Infinity Lab Poroshell 120 EC-C18; 50 × 3 mm ID, 2.7 μm particle size (Agilent 6999975-302T), was selected for analyses in most years because it provides optimal separation of individual compounds. Lipid content in egg samples was determined gravimetrically. Details about chemical analyses have been described previously.^19^

### Quality Assurance/Quality Control

Quality assurance and quality control (QA/QC) was assessed in two ways. In earlier years, accuracy was assessed by analyzing either an aliquot of an in-house double-crested cormorant egg pool containing 4–5 detectable PFASs and/or duplicates of randomly selected egg samples. In all other years, QA/QC was assessed by analyzing a “clean” (i.e., absence of PFASs) organic chicken egg pool that was spiked with the full suite of PFASs plus IS solution (MeOH, 2000 ng/mL containing 9 compounds: MPFBA, MPFHxA, MPFOA, MPFNA, MPFDA, MPFUdA, MPFDoA, MPFHxS, MPFOS from Wellington Labs, Part# MPFAC-MXA). The recoveries for the PFASs were within the acceptable ranges (80–120%), demonstrating good method accuracy. Concentrations of PFASs in samples were calculated using the internal standard method. The method detection limits (MDLs) were calculated by multiplying a Student’s *t* value by the standard deviation (between 8 replicates, matrix spike at low concentrations). Within the QuEChERS procedure, MDLs were between 0.01 and 0.05 ng/g (ppb).

### Stable Isotope Analyses

In most years, stable isotope analyses (SIAs) of carbon (δ^13^C) and nitrogen (δ^15^N) were carried out using the same pooled egg homogenates as used for chemical analyses. SIAs were performed at the G. G. Hatch Stable Isotope Laboratory in Ottawa, ON, or the Davis Stable Isotope Facility at the University of California, as described previously.^16,19^

### Statistical Analyses

All statistical analyses were carried out in R (V4.1.2) and were performed on compounds that had detectable concentrations in >50% of all egg samples in each species. Compounds in samples that were less than the MDLs (or not detected; nd) were summarized for each species and year using a Kaplan–Meier (KM) model with the *NADA*(20) and *NADA2*(21) packages, and set to 0 for calculating Σ_17_PFASs, Σ_4_PFSAs, and Σ_13_PFCAs. Changes in the percent contributions of individual PFASs to Σ_17_PFASs, Σ_4_PFSAs, and Σ_13_PFCAs were analyzed using a Spearman Rank Correlation. All PFAS concentrations are expressed in ng/g wet weight (ww) and were ln(log)-transformed prior to statistical analyses.

To assess whether PFAS concentrations decreased over time, we fitted a set of Generalized Additive Models (GAMs) with a Gaussian distribution and identity link function for each species using the *mgcv* package^22^ in R. Each PFAS/ΣPFAS group was analyzed separately as the dependent variable with year and dietary tracers (δ^13^C and δ^15^N) as continuous variables, and breeding colony location as a fixed effect to account for potential spatial differences in contamination across the south, mid, and north coasts. To account for nonlinearity, we entered the year as a smoothed term in the GAMs using thin-plate splines (*ts*) and adapted the smoothing parameter (*k*) to avoid overfitting. Predicted values were extracted from the GAMs and used for plotting the fitted trend lines for each PFAS/ΣPFAS group. We performed model selection using Akaike Information Criterion (adjusted for small sample sizes, AIC_C_), ΔAIC_C_ (change in AIC_C_), AIC_W_ (AIC_C_ weight), and *R*^2^ using the *MumIn* package^23^ and selected the model with the lowest AIC_C_ as the final model (Tables S1–S4). For non-detects, we fitted a set of models for each species by first using the lowest AIC before interpreting outputs (*p* value, *R*^2^) from a censored multiple regression model with the *NADA2* package^21^ in R. Temporal trends for censored PFASs were plotted using a Kendall’s Tau (τ) test of change and an Akritas-Theil-Sen (ATS) regression line.^20,21^ Doubling/halving times for PFASs were estimated with *t*_1/2_ = ln(2)/*m*, where *m* is the slope of the ln transformed concentration versus time following first-order kinetics.

Stable isotopes of egg δ^15^N and δ^13^C values were compared among species and breeding colonies using linear models with each stable isotope analyzed separately as the dependent variable and species and breeding colony location as fixed effects. Relationships between isotopes and PFAS concentrations were examined across species using Generalized Additive Mixed Models (GAMMs) with year as a nonlinear term and breeding colony location as a random effect. Isotopic niche parameters (i.e., convex hulls) were generated using the *SIBER* package.^24^ One outlier was removed (LSPE, Cleland Island 2006, δ^13^C = −16.9, δ^15^N = 14.8) from SIAs. The statistical significance of *p* values for all models was assessed at α = 0.05.

## Results and Discussion

### Concentrations and Composition Profiles of PFASs

**Figure 1:**
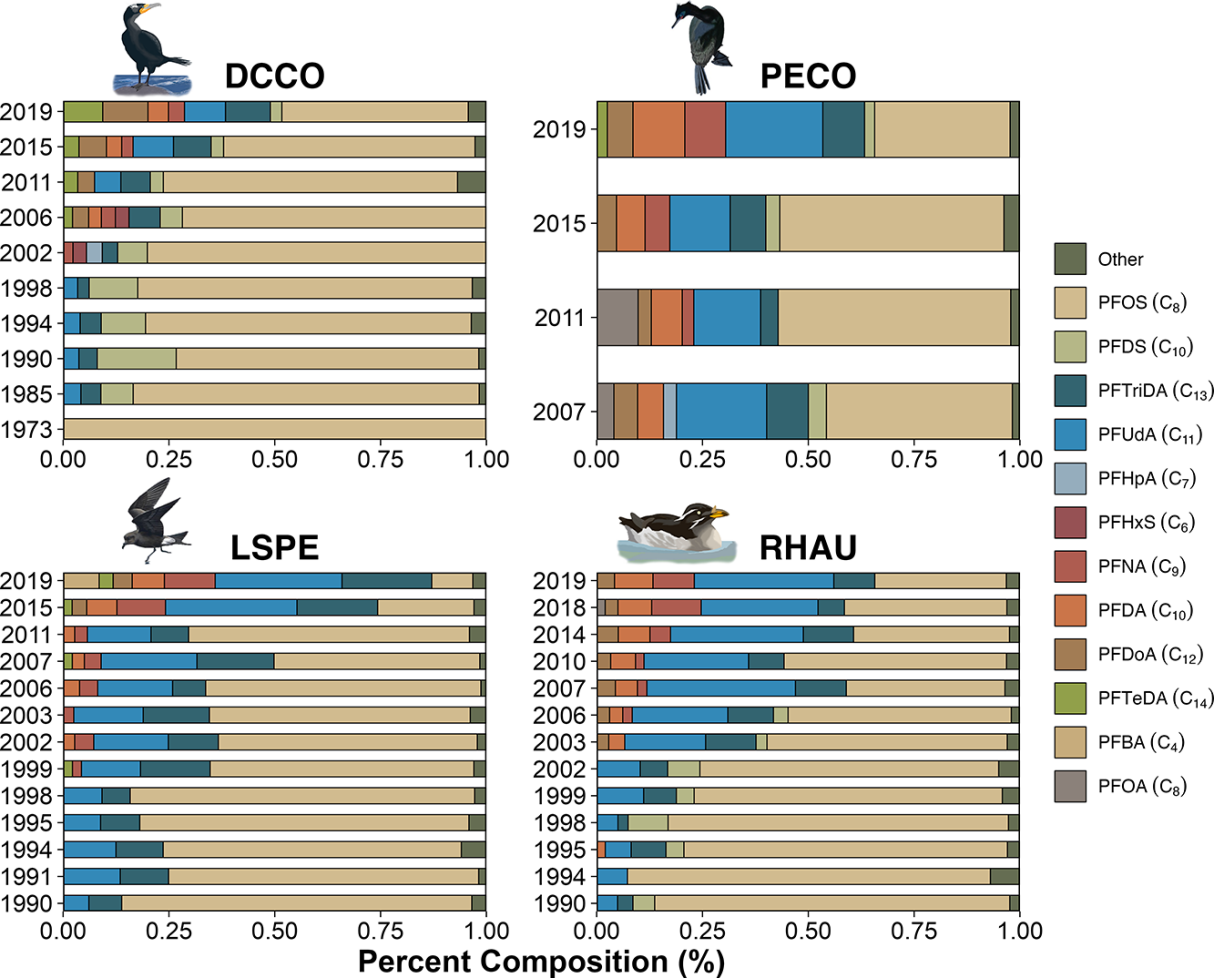
Percent contribution (%) of major perfluoroalkyl substances (PFASs) sampled from 1973 to 2019 in the eggs of double-crested cormorants (DCCOs), pelagic cormorants (PECOs), Leach’s storm petrels (LSPEs), and rhinoceros auklets (RHAUs) from the Pacific coast of BC.

PFOS was consistently and by far the most abundant PFSA detected (Figure 1), constituting >79 to 89% of Σ_4_PFSAs in the eggs of cormorants and >89 to 94% in the eggs of auklets and storm petrels across all years. Comparatively, mean concentrations of PFOS were highest in RHAU eggs (286 ng/g ww; 1998) > DCCO eggs (60.4 ng/g; 1994) > LSPE eggs (52.7 ± 4.7 ng/g; 2011) > PECO eggs (39.3 ± 15.7 ng/g; 2011). The lowest quantifiable PFOS concentrations were all measured in 2019 (Table 1). PFDS showed a stronger presence in the eggs of cormorants, with concentrations ranging from 0.1 to 8.2 ng/g ww, comprising <11 to 21% of Σ_4_PFSAs, while those in auklet and storm-petrel eggs ranged up to 34 ng/g, comprising <3 to 11% of Σ_4_PFSAs, in each year. PFBS was detected in only 2 DCCO egg pools (<0.3 ng/g; 2011), whereas PFHxS was detected intermittently (<1 ng/g), comprising <6% in LSPE eggs, <5% in DCCO eggs, <3% in RHAU eggs, and <1% in PECO eggs, of Σ_4_PFSAs across years. These findings are consistent with a study from the same region where PFBS and PFDS were highly dominant among PFSAs in cormorant and great blue heron egg pools from 1973 to 2012^19^ and likely reflect the relative proximity of those species’ colonies to contaminated sites and urbanized areas (e.g., Victoria, Metro Vancouver, Puget Sound) with industrial inputs and large municipal populations.^25,26^

**Table 1:** Concentrations of Major Perfluoroalkyl Substances (Arithmetic Mean ± Standard Error; ng/g Wet Weight) in Pacific Seabird Eggs Collected from the Breeding Colonies Located in S Figure 1^a^^,^^b^

species	sample size	year	% moisture	% lipid	PFNA	PFUdA	PFTriDA	PFOS	Σ_4_PFSAs	Σ_13_PFCAs	Σ_17_PFASs
double-crested cormorant (*Nannopterum auritum*)	1 pool, *n* = 8	1973						9.60	9.60		9.60
	1 pool, *n* = 14	1998	84.1	6.1	0.39	1.90	1.50	43.8	51.0	4.46	55.4
	5 pools, *n* = 3	2019	84.0	3.39 ± 0.3	0.45 ± 0.1	1.15 ± 0.1	1.26 ± 0.3	5.23 ± 1.1	5.75 ± 1.2	6.04 ± 1.5	11.8 ± 2.6
pelagic cormorant (*Urile pelagicus*)	5 pools, *n* = 3	2007	84.5 ± 0.2	5.36 ± 0.3	ND	6.28 ± 1.1	2.90 ± 0.5	12.9 ± 1.9	14.2 ± 2.1	13.1 ± 2.4	27.3 ± 4.5
	5 pools, *n* = 3	2011	83.8 ± 0.6	3.20 ± 0.2	1.95 ± 1.0	11.3 ± 3.8	3.04 ± 0.3	39.3 ± 16	40.4 ± 15	24.7 ± 8.4	65.0 ± 24
	5 pools, *n* = 3	2019	83.6 ± 0.13	3.20 ± 0.5	1.26 ± 0.2	3.00 ± 1.0	1.29 ± 0.4	4.18 ± 1.0	4.52 ± 1.1	8.44 ± 2.4	13.0 ± 3.5
rhinoceros auklet (*Cerorhinca monocerata*)	11 pools, *n* = 3	1990	70 ± 0.5	11.8 ± 0.2	0.45 ± 0.1	4.53 ± 0.01	3.34 ± 0.02	77.0 ± 8.7	82.1 ± 11	9.65 ± 0.1	91.7 ± 11
	15 pools, *n* = 2–3	2010	66.5 ± 2.5	11.3 ± 0.7	2.58 ± 0.2	31.6 ± 2.2	10.7 ± 0.7	67.3 ± 7.5	69.2 ± 7.6	58.8 ± 3.7	128 ± 9.2
	15 pools, *n* = 3	2018	69.2 ± 0.4	12.5 ± 0.5	7.0 ± 0.6	17.8 ± 0.8	4.29 ± 0.2	22.8 ± 1.8	23.6 ± 1.9	38.2 ± 1.7	61.8 ± 3.2
Leach’s storm petrel (*Hydrobates leucorhous*)	1 pool, *n* = 15	1990	70.9	11.8	0.46	2.24	2.86	30.5	30.7	6.18	36.8
	16 pools, *n* = 2–3	2011	71.9 ± 0.6	11.2 ± 0.3	2.37 ± 0.2	11.9 ± 0.5	7.06 ± 0.5	52.7 ± 4.7	52.8 ± 4.8	25.9 ± 1.0	78.7 ± 5.1
	15 pools, *n* = 3	2019	69.4 ± 0.43	11.5 ± 0.45	2.02 ± 0.1	5.04 ± 0.2	3.57 ± 0.1	1.63 ± 0.1	1.70 ± 0.1	13.6 ± 0.6	15.3 ± 0.6

a “ND” = Not detected based on the method detection limit(MDL). “–” = Not analyzed.

b For conciseness, concentrations shown are representative of the earliest, intermediate, and recent years of reporting for that species.

Whether local point sources are playing a major role in the spatial distribution of PFOS in our species is unclear since eggs collected from relatively remote colonies (e.g., Cleland Island) had up to 3.5× higher concentrations of PFOS, on average, than eggs/species sampled from more urban colonies. PFOS, its salts, and precursors were never manufactured in Canada.^27^ As a result, PFOS use on Vancouver Island was likely limited to indirect sources (pulp/paper mills, indoor use), along with AFFF applications at fire stations and possibly Tofino-Long Beach Airport (∼25 km from Cleland Island). Increased shoreline development and untreated wastewater from The District of Tofino via Duffin Passage may have also been responsible for the greater PFOS contamination around Clayoquot and Barkley Sounds.^28,29^ Alternatively, auklets and storm petrels breeding off the west coast of Vancouver Island may have been exposed to PFASs via LRT from Asia, including eastern coastal provinces in China where many PFASs are still manufactured and used to the present day.^6,9,19^ The leaching and ingestion of PFASs from floating plastics in connection with the Great Pacific Garbage Patch may have additionally influenced exposure in some species, although further studies are needed to confirm these findings. Interspecific differences in PFOS patterns in our species were also a function of several likely biological and toxicokinetic factors, such as diet and origin of prey items (at both individual and colony levels), biotransformation of PFOS precursors (*N*-EtPFOSA → PFOSA → PFOS), migratory status, egg lipid/protein content, and selective maternal transfer.^27,30−32^

Relative compositions of PFCAs were similar among species (Figure 1). In PECO eggs, PFUdA was the most frequently detected PFCA, with concentrations ranging from 1.2 to 26 ng/g, comprising <53% of Σ_13_PFCAs, while those for PFDA, PFDoA, and PFTriDA ranged from 0.3 to 13 ng/g, and represented <30% of Σ_13_PFCAs in each year. In DCCO eggs, the primary PFCAs were PFTriDA, PFUdA, PFTeDA, and PFDoA, averaging <45, <43, <33, and <24% of ΣPFCAs, respectively, in each year. Concentrations of these PFCAs ranged from 0.5 to 4, nd-3.1, nd-3.1, and nd-2.4 ng/g, respectively. Similarly, PFUdA and PFTriDA were the dominant PFCAs in RHAU eggs (<62, <44% of ΣPFCAs, respectively) and LSPE eggs (<61, <47% of Σ_13_PFCAs, respectively). Concentrations of these PFCAs in auklet and storm petrel eggs ranged from 2.2 to 54.4 and 2.1 to 16 ng/g, respectively. These PFCA patterns are virtually identical to those reported by Miller et al. in the same species and region during the 1990s–2000s, and by Gebbink et al. in glaucous-winged gull eggs from Vancouver Island (Mandarte and Florencia) in 2008, with marine (and some terrestrial) prey having been the likely source of exposure to gulls. Short-chain PFCAs, such as PFBA, PFPeA, and PFHxA were unquantifiable or detected at very low concentrations (<1.6 ng/g) in the eggs of our species in all years, likely owing to their high solubility and low bioaccumulation potential in most aquatic biota.^11,35,37^

Relative compositions of PFASs varied by sampling year, with both long- and odd-chain PFCAs dominating the profile in recent years (Figure 1). Relative proportions of PFOS in Σ_17_PFAS statistically decreased over time in the eggs of all species (*p* < 0.05) but pelagic cormorants (*p* > 0.1), which may have been an artifact of small sample size. Among the PFCAs, there was a statistical increase in the contribution of PFUdA, PFTriDA, PFNA, and PFDA to the PFAS profile in DCCO, RHAU, and LSPE eggs (*p* < 0.05). The largest percent changes were observed for PFUdA in RHAU and LSPE eggs where relative proportions statistically increased from <14% in the early 1990s to >30% in the recent year of sampling (*r*_s_ = 0.91; *p* < 0.001 and *r*_s_ = 0.89; *p* < 0.001, respectively), while those in DCCO and PECO eggs remained stable (*r*_s_ = 0.53; *p* = 0.11 and *r*_s_ = −0.4; *p* = 0.6, respectively) over time. Relative proportions of PFTriDA in Σ_17_PFAS also statistically increased over time in the eggs of double-crested cormorants (*r*_s_ = 0.79; *p* < 0.01) and storm petrels (*r*_s_ = 0.61; *p* < 0.05) but not in the other two species (*p* > 0.1).

### Temporal Trends

**Figure 2:**
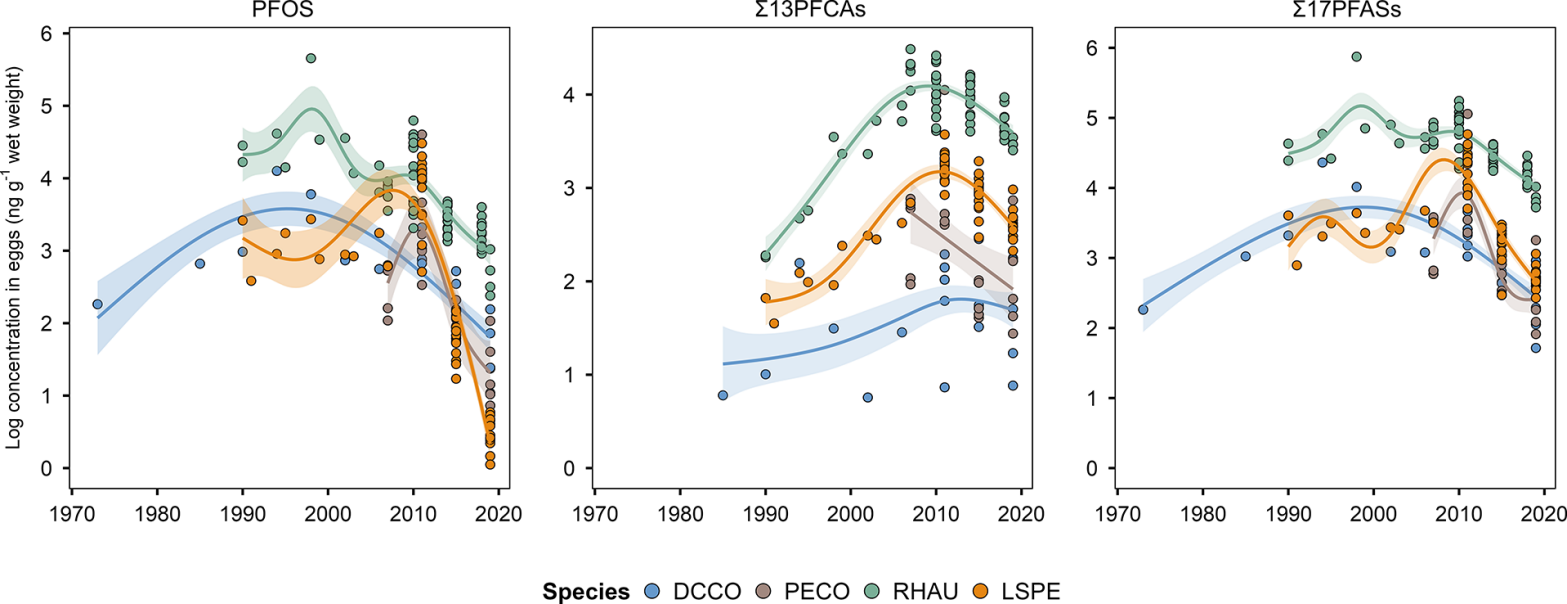
Temporal trends of PFOS, Σ_13_PFCAs (PFBA, PFPeA, PFHxA, PFHpA, PFOA, PFNA, PFDA, PFUdA, PFDoA, PFTriDA, PFTeDA, PFHxDA, and PFODA), Σ_17_PFASs (PFBS, PFHxS, PFOS, PFDS, PFBA, PFPeA, PFHxA, PFHpA, PFOA, PFNA, PFDA, PFUdA, PFDoA, PFTriDA, PFTeDA, PFHxDA, and PFODA) in the eggs of double-crested cormorants (DCCOs), pelagic cormorants (PECOs), rhinoceros auklets (RHAUs), and Leach’s storm petrels (LSPEs) sampled from the Pacific coast of BC, Canada. Dots represent annual concentrations in egg pool samples. Trend lines and 95% prediction intervals (shaded) are fitted using GAM models.

Concentrations of PFOS, PFDS (Figure S2), and Σ_4_PFSAs in DCCO egg pools increased for over two decades and peaked between 1994 and 1998 before decreasing by up to ∼74% from 2011 to 2019 (Figure 2; *p* < 0.001). Although concentrations of PFOS and Σ_4_PFSAs were lower in 2007 than in 2011 in PECO eggs, the longer-term trend from DCCO eggs clearly shows an earlier peak, with PECO eggs gradually declining from their peak by ∼89% by 2019 (Figure 2; *p* < 0.001). These trends are consistent with earlier reports on these PFSAs in DCCO eggs from Mandarte Island sampled from 1973 to 2011^19^ and indicate further decreasing trends with a statistically determined breakpoint in 2011. Our PFOS halving time of 6.90 years in DCCO eggs showed a ∼14-fold decrease relative to the time reported previously (96.3 years).^19^ The PFOS halving time for PECO eggs was 5.62 years. These results clearly demonstrate a slowing accumulation of PFOS in cormorant eggs from the Salish Sea. The statistical effect of δ^13^C on PFOS trends in cormorant eggs (Tables S1 and S2) further suggests a diminishing PFOS influx at the Pacific coast concomitant with more nearshore and inland feeding. Concentrations of censored PFSAs (e.g., PFHxS) in cormorant eggs generally declined over time (Figures S6 and S7).

Our most parsimonious description describing temporal trends in PFAS concentrations in auklet and storm-petrel egg pools was one that included effects for year and/or location, although, other models were equivalent based on the ΔAIC_C_ (Tables S3 and S4). Concentrations of PFOS and Σ_4_PFSAs in storm-petrel eggs from all three colonies were relatively constant until the early 2000s and were then followed by a pronounced decrease (Figure 2; *p* < 0.001) from 2011 to 2019 (↓97%) with a PFOS half-life of ∼2.57 years. In the case of PFOS in auklet eggs, two consecutive decreases occurred from 1998 to 2007 (↓84%) and 2010 to 2019 (↓78%) with a half-life of ∼7.76 years (Figure 2; *p* < 0.001). Concentrations of other PFASs in auklet and storm-petrel eggs generally declined over time or showed no major trends (Figures S4, S8, and S9).

The PFOS trends observed in our species moderately align with the voluntary phase-out of PFOS and its C_8_ precursors by 3M circa 2000–2003.^8^ Declining trends in egg PFOS concentrations following the 3M phase-out have also been reported in double-crested cormorants from San Francisco Bay;^36^ northern gannets from the United Kingdom;^37^ tawny owls from Norway;^38^ peregrine falcons from Sweden;^39^ Audouin’s gulls and yellow-legged gulls from the Mediterranean;^40^ herring gulls from the Laurentian Great Lakes^30^ and Norway;^32^ and common murres from the Baltic Sea;^41^ however, trends have not been universally consistent.^26,27,42−46^ The lagged response of PFOS in birds and other wildlife has been largely attributed to increased production of PFOS and its precursors in China post-2003 (thus offsetting progress made by some North American, European, and Asian countries),^6,9,27^ differences in transport pathways (e.g., rapid atmospheric transport vs slow oceanic transport),^38,73^ as well as differences in biotransformation/degradation rates of PFOS precursors in different environmental media.^30,36^ Spatial and temporal trends may have also been influenced, to an extent, by site-specific environmental conditions. For example, long residence times and enclosed inland waters in the Strait of Georgia (relative to some offshore areas) may have impeded the dilution of PFOS and other contaminants,^16,47,48^ thus delaying the onset of decreasing trends in some of our species.

Concentrations of PFNA, PFTriDA (Figure S2), and Σ_13_PFCAs (Figure 2) in DCCO eggs were relatively stable over time (*p* > 0.5), while those for PFDoA and PFTeDA (Figure S6) slightly increased (*p* < 0.01) during the same period, possibly reflecting changes in detection limits and differences in contamination of benthic versus midwater specialists. In PECO eggs, concentrations of PFDA, PFUdA (Figure S3), and Σ_13_PFCAs (Figure 2) peaked in 2011 before decreasing (*p* < 0.001) in the later period. Meanwhile, PFNA in PECO eggs showed a marginal increase (Figure S7; *p* < 0.05), while PFOA, PFDoA, PFTeDA, and PFTriDA saw a decline and remained more or less constant (Figure S7; *p* < 0.05). Concentrations of PFDA, PFUdA, PFDoA, PFTriDA (Figures S4 and S5), and Σ_13_PFCAs (Figure 2) in auklet and storm-petrel eggs exhibited a trajectory comprised of linear increases and second-order declines (*p* < 0.001) in recent years, suggesting that the rate of uptake of PFCAs in these species is slowing or leveling off. The temporal rise in Σ_13_PFCAs in auklet and storm-petrel eggs was primarily caused by increasing concentrations of PFNA (Figures S4 and S5; *p* < 0.001), with doubling times being 7.57 and 10.97 years, respectively. Environmental loadings of PFNA have been linked to the production and use of ammonium perfluorononanoate (AFPN) in Japan, and its products (e.g., Surflon S-111), which often contain C_4_-C_13_ PFCA homologs as impurities in various amounts.^4,12^

The sharp decline in PFCA concentrations in the eggs of our species and sampling region after 2010 is consistent with the phase-out and elimination of long-chain PFCAs by the US EPA PFOA Stewardship Program^11,38^ and the ECCC and Health Canada PFCA Environmental Performance Agreement, which took effect from 2010 to 2015.^19^ However, some companies may have chosen not to participate in these programs, and it is possible that such PFCA phase-outs have not resulted in sufficient time to detect decreasing trends in other species and geographic regions. For example, increasing trends for C_9_-C_13_ PFCAs were reported in the eggs of tawny owls from 1986 to 2019 in Norway^38^ and in black-tailed gulls from 2015 to 2019 in Korea.^45^ Miller et al. proposed that the increasing trends of C_9_-C_13_ PFCAs in seabird eggs may be attributed to direct sources (i.e., manufacturing and use) in Asia and indirect sources (i.e., atmospheric degradation of precursors), although the relative importance of the two pathways in terms of influencing spatial/temporal trends of PFCAs in the eastern North Pacific Ocean is still unclear.

The eastern North Pacific Ocean is exposed to high inputs of oceanic and atmospheric PFCAs from the Bering Strait,^49^ presumably via the Oyashio Current and Kuroshio Current, both of which run eastward off the coast of Japan, eventually bifurcating off the southern BC coast into the southward-flowing California current and northward-flowing Alaska current. Yet, oceanic transport is a relatively slow process and it can take several years for PFCAs from direct emissions to reach some North American regions.^50,51^ Odd-number chain-length PFCA patterns, such as those observed in our species, are now thought to be produced via the process of atmospheric degradation of precursors, as documented through multiple lines of evidence. First, PFCA precursors (e.g., 8:2, 10:2 FTOHs) are volatile and have an atmospheric half-life of ∼20 days, allowing them to undergo rapid atmospheric LRT to remote regions.^52^ Second, PFCA precursors contain even carbon-chain homologs and can degrade in the atmosphere (and in biota) to yield odd- and long-chain PFCAs.^4,12,52^ Third, emissions of PFCA atmospheric precursors and C_9_-C_13_ PFCAs have been increasing since 2003,^6^ thus facilitating their hemispheric distribution. Finally, many of the auklet and storm-petrel colonies from our region are upwind from any industry and have limited land-based inputs,^16,53^ further supporting the hypothesis of atmospheric degradation as an important transport mechanism. To take a case in point, the 7:3-fluorotelomer carboxylic acid (7:3 FTCA) precursor was the most abundant PFAS (>41%) in Southern Resident and Transient killer whale tissue samples collected between 2006 and 2018 along the BC coast,^54^ with many of those sampling sites overlapping with our own.

The preferential bioaccumulation of C_10_-C_15_ PFCAs (e.g., PFUdA, PFTriDA) in seabird eggs is also thought to be in part a function of specific yolk proteins, such as vitellogenin and very-low-density lipoproteins (VLDL), synthesized in the liver and maternally transferred into the egg yolk.^15,31^ In contrast to double-crested cormorant and pelagic cormorant eggs, auklet, and storm-petrel eggs have relatively higher egg lipid percentages and could equally have higher egg protein content, consequently resulting in higher sorption capacities for some PFCAs.^55^

### Species Differences in Relation to Habitat Use and Marine Input (δ^13^C)

**Figure 3:**
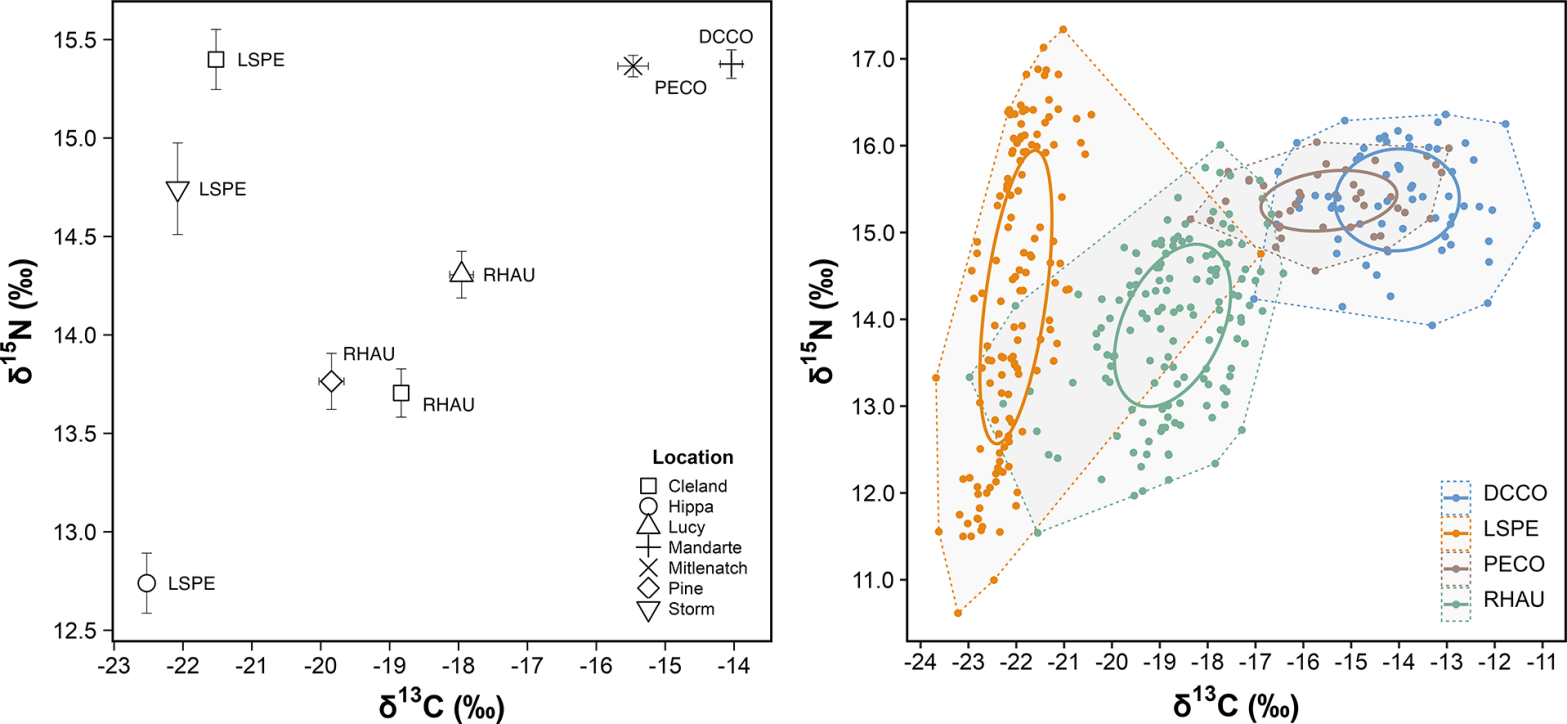
Left plot: Mean δ^15^N and δ^13^C isotope biplot. DCCO, double-crested cormorant; PECO, pelagic cormorant; RHAU, rhinoceros auklet; LSPE, Leach’s storm-petrel. Error bars represent the standard error. Right plot: Population niche width and dietary overlap of δ^15^N and δ^13^C isotope signatures of seabird eggs. Circles represent the maximum likelihood standard ellipses (sample size corrected area). Dashed lines and shaded regions represent the convex hull total areas.

Egg δ^13^C values varied among species (*F*_3,382_ = 1139; *p* < 0.001), with average δ^13^C ranging from −22.0‰ (±0.1‰), −18.7‰ (±0.1‰), −15.5‰ (±0.2‰), and −14.0‰ (±0.2‰) in the eggs of Leach’s storm petrels, rhinoceros auklets, pelagic cormorants, and double-crested cormorants, respectively, across all locations and years (Figure 3). δ^13^C values slightly varied among the three auklet colonies (*F*_2,142_ = 34.0; *p* < 0.001) with the lowest mean values observed at Pine Island (−19.8 ± 0.2‰), followed by Cleland Island (−18.8 ± 0.1‰), and Lucy Island (−18.0 ± 0.2‰). δ^13^C also differed among storm-petrel colonies (*F*_2,137_ = 40.5; *p* < 0.001) with Cleland Island eggs more enriched in δ^13^C (−21.5 ± 0.1‰) than eggs from more northerly colonies at Storm Island (−22.1 ± 0.4‰) and Hippa Island (−22.5 ± 0.4‰).

**Figure 4:**
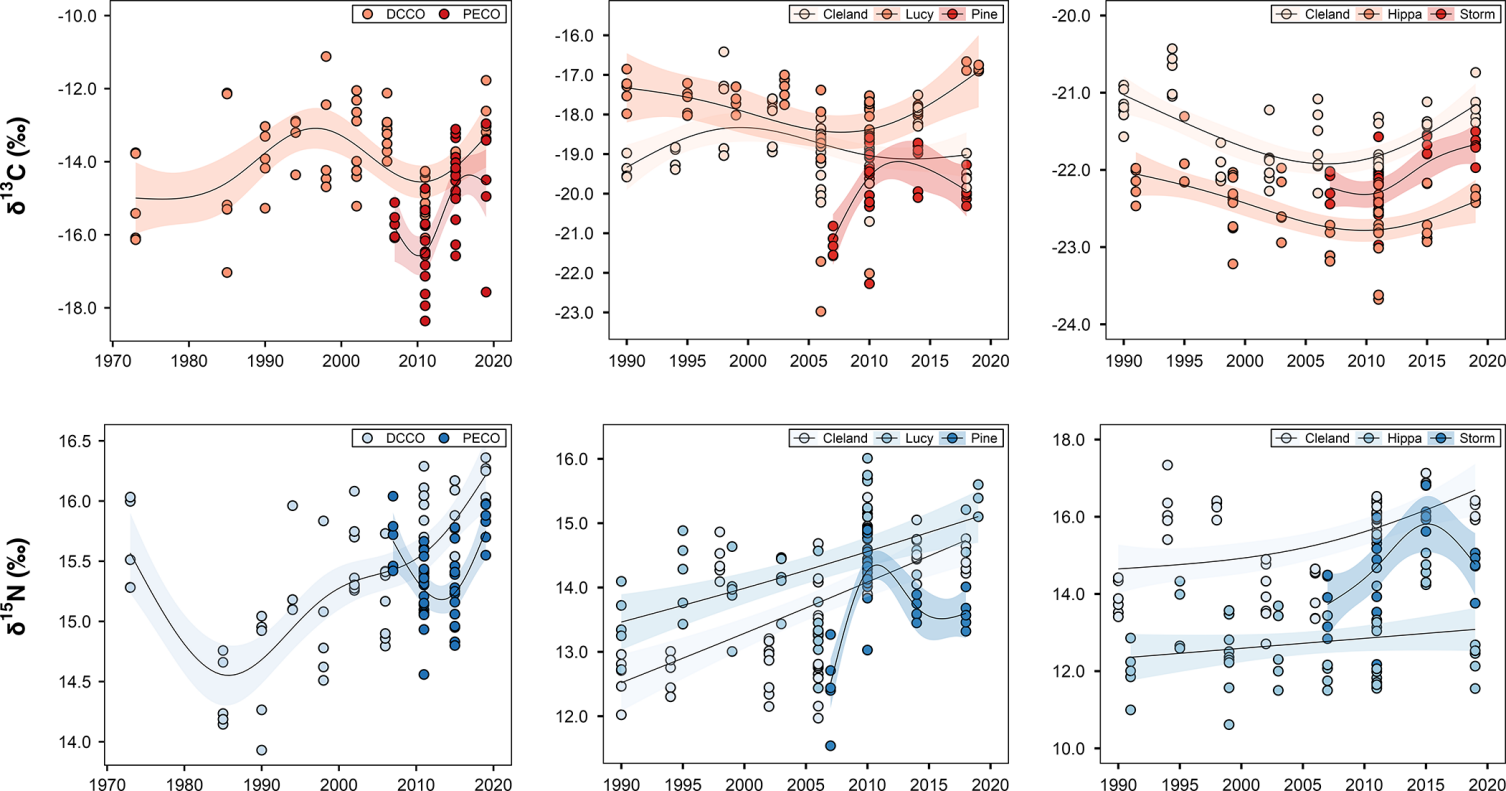
Temporal trends of δ^15^N and δ^13^C values in the eggs of double-crested cormorants (DCCOs) and pelagic cormorants (PECOs; left plots), rhinoceros auklets (RHAUs; middle plots), and Leach’s storm petrels (LSPEs; right plots). Data points represent annual isotopic signatures in egg pool samples. Trend lines and 95% prediction intervals (shaded) are fitted using GAM models.

For the two cormorants, egg δ^13^C values spanned a wide range (∼5‰) and presented oscillating variations before increasing toward the end of the study period (Figure 4; *p* < 0.001). Cormorants forage widely over the Salish Sea; however, our increasing δ^13^C values coupled with the declining δ^34^S values reported previously^56^ strongly suggest that some cormorants from our study region fed in more nearshore and inland environments. By contrast, δ^13^C values decreased and subsequently increased in the eggs of auklets and storm petrels (Figure 4; *p* < 0.001). These trends closely mirror those previously reported for the same species and region,^16,18,19,57^ and suggest continuing differences in habitat use and feeding behavior among laying females, with auklets on or near the continental shelf and storm-petrels in more offshore pelagic areas. The Suess effect (i.e., the systematic decline in δ^13^C due to carbon emissions) likely played a negligible role in our SIAs due to the relatively short time span of our data.^19,56^

Values for δ^13^C across species were nonlinearly correlated with lnΣ_17_PFASs (*t*_158_ = −2.96; *p* < 0.01), but not PFOS (*t*_156_ = 0.73; *p* = 0.47), Σ_4_PFSAs (*t*_156_ = 1.12; *p* = 0.26), or short-chain ΣPFASs (*t*_101_ = −0.46; *p* = 0.64). Marine input and urbanization (approximated by δ^13^C) are often strong predictors of emergent contaminant concentrations^53,58−60^ and could partly explain why seabirds foraging in different marine habitats, such as coastal (higher δ^13^C) versus offshore (lower δ^13^C), have different PFAS burdens. The absence of a statistical relationship between δ^13^C and PFOS/Σ_4_PFSAs across our species may reflect the ubiquitous nature of PFOS in the eastern North Pacific with multiple sources/exposure pathways, consequently homogenizing exposure and leading to a spatial averaging of PFAS bioaccumulation over the region.^61^

Values for δ^13^C across species were also nonlinearly correlated with lnΣ_13_PFCAs (*t*_157_ = −5.90; *p* < 0.001) and long-chain ΣPFASs (*t*_158_ = −2.99; *p* < 0.01). Elliott et al. found that δ^13^C values were negatively associated with PFUdA and PFTriDA (as well as polybrominated diphenyl ethers, hexachlorobenzene, and β-hexachlorocyclohexane) in rhinoceros auklet eggs collected from the same breeding colonies sampled in the present study, with PFTriDA levels slightly increasing with wintering latitude. Auklets from our long-term monitoring colonies typically overwinter in coastal neritic waters off Vancouver Island, California, Mexico, and Alaska,^59,62^ which could give auklets access to PFAS-contaminated prey year-round, resulting in carryover from wintering exposure to deposition in eggs.^19^ Similarly, Leach’s storm petrels tracked from Vancouver Island had low feather δ^13^C values (mean = −19‰) that were concomitant with large foraging ranges extending beyond the Canadian Exclusive Economic Zone (EEZ), with some storm petrels traveling up to 1600 km from their colony during the breeding season and up to 6700 km from their colony during the nonbreeding season.^63^ That is consistent with more offshore habitat use throughout the annual cycle and could place some migratory Procellariiformes closer to historical/ongoing sources of PFCAs in the Northern Hemisphere.^64^ Other studies have likewise failed to find strong evidence linking δ^13^C values to PFCA concentrations in seabird eggs,^19,65^ possibly due to δ^13^C spanning a wide range of species, aquatic/terrestrial habitats, and time periods, thus confounding any relationships.

### Species Differences in Relation to Dietary Exposure and Trophic Level (δ^15^N)

Egg δ^15^N varied among species (*F*_3,382_ = 52.4; *p* < 0.001), with average δ^15^N ranging from 13.93‰ (±0.1‰), 14.26‰ (±0.1‰), 15.37‰ (±0.1‰), and 15.38‰ (±0.1‰) in the eggs of rhinoceros auklets, Leach’s storm petrels, pelagic cormorants, and double-crested cormorants, respectively, across all locations and years (Figure 3). δ^15^N varied among auklet colonies (*F*_2,142_ = 7.01; *p* < 0.01) with mean δ^15^N higher in eggs from Lucy Island (14.31 ± 0.1‰) than in eggs from Cleland Island (13.71 ± 0.1‰) and Pine Island (13.76 ± 0.1‰). δ^15^N also varied among storm-petrel colonies (*F*_2,137_ = 77.9; *p* < 0.001) with Cleland Island eggs more enriched in δ^15^N (15.40 ± 0.2‰) than eggs from Storm Island (14.74 ± 0.2‰) and Hippa Island (12.74 ± 0.2‰).

Overall, δ^15^N trends followed a quadratic relationship in cormorant eggs (Figure 4; *p* < 0.001), while those in auklet and storm-petrel eggs gradually increased during the study period (Figure 4; *p* < 0.001). While these trends would traditionally be interpreted as an increase in trophic level, it is important to note that nitrogen at the base of food webs (baseline δ^15^N) can vary across different spatial scales and time periods,^34,65,66^ thus influencing bulk δ^15^N (or whole-body tissue) values in higher consumers.^67^ Amino acid-specific SIAs offer an opportunity to avoid those problems because some amino acids increase in δ^15^N (trophic amino acids) due to fractionation at lower trophic levels, while others show little change in δ^15^N (source amino acids) due to minimal transamination at higher trophic levels.^65^

Values for δ^15^N were measured in source and trophic amino acids in a subset of seabird eggs sampled from the present study.^16,67^ Those analyses revealed that ∼50% of the variation in δ^15^N for trophic amino acids was associated with variation in source amino acids, thus obscuring trends in bulk δ^15^N values and trophic position. Egg data adjusted for baseline values (δ^15^N_trophic–source_) revealed that storm petrels and auklets sampled from the same breeding colonies as the present study occupied relatively high trophic positions, while cormorants breeding on Mandarte and Mitlenatch Islands had intermediate trophic positions.^16^ Taken together, our SIAs suggest that cormorants breeding on Mandarte and Mitlenatch Islands are feeding on a combination of midwater prey and increasingly more benthic prey, possibly due to declines in forage fish (e.g., herring) abundance.^56^ Auklets from our monitoring colonies appear to be feeding on an exclusive diet of fish (e.g., sandlance) and/or higher trophic level zooplankton.^57,62^ The latitudinal gradient in δ^15^N for storm-petrel eggs suggests a diverse diet of crustaceans/euphausiids,^68^ myctophid fish,^65^ and other omega-3 rich prey,^69^ and perhaps, much more spatial heterogeneity in baseline δ^15^N caused by denitrification and advection of eastern Tropical Pacific water masses.^70,71^

Values for δ^15^N across species were nonlinearly correlated with lnΣ_17_PFASs (*t*_158_ = −5.08; *p* < 0.001), long-chain ΣPFASs (*t*_158_ = −5.14; *p* < 0.001), Σ_4_PFSAs (*t*_156_ = −2.88; *p* < 0.01), Σ_13_PFCAs (*t*_157_ = −5.73; *p* < 0.001), and PFOS (*t*_156_ = −3.06; *p* < 0.01), but not short-chain ΣPFASs (*t*_101_ = −0.51; *p* = 0.61). The correspondence between different isotope values and annual fluctuations in PFAS levels suggests that other factors beyond emission history, such as diet and feeding habits, may have influenced temporal trends of PFASs in the northeast Pacific, and that these trends are not necessarily in a consistent direction we would expect from solely regulation. Long-chain PFASs are characterized by their high *K*_OW_ (>10^5^–10^9^) and *K*_OA_ (>10^6^) values and are more bioaccumulative than short-chain PFASs,^72^ consequently increasing their biomagnification potential within food webs. Diet is also a major route of exposure, and it is therefore not surprising that previous studies alongside the current study found associations between δ^15^N and PFASs, in both aquatic^30,34,64^ and terrestrial^60,73,74^ systems. However, variation in baseline δ^15^N across seabird colonies may have created a spurious relationship between PFASs and bulk δ^15^N, while masking a relationship between other PFASs and δ^15^N,^19,75,76^ a pattern consistent with legacy POPs such as dichlorodiphenyltrichloroethane (DDT) and polychlorinated biphenyls (PCBs).^16,65^ Thus, bulk δ^15^N may not be a reliable proxy for the relative trophic position and biomagnification propensity of PFASs in seabird food webs. The use of different dietary tracers and amino acid-specific isotopes could provide a more refined estimate of diet and improve future studies.

### Toxicological Implications

Studies have shown that although PFOS is often the dominant PFAS detected in bird eggs, it may not be acutely toxic at concentrations up to 3500 ng/g.^7,77−79^ The predicted no-effect concentration (PNEC) and toxicity reference value (TRV) derived by Newsted et al. for PFOS in egg yolk (based on acute and chronic laboratory exposure in northern bobwhite quail and mallard) are 1000 and 1700 ng/g, respectively. Concentrations of PFOS in our egg pools were several orders of magnitude below these thresholds. Molina et al. estimated a lowest-observed-adverse effect level (LOAEL) of 100 ng/g for PFOS in eggs, which was exceeded in some of our auklet egg pools (1994, 1998, 2010), suggesting potential effects on hatchability and pipping success in those years. However, given that PFOS levels in our auklet (and other) egg pools in recent years (2018/9) were all relatively low (<37 ng/g ww) and well below the LOAEL, these findings are not of toxicological concern. Tartu et al. found that plasma corticosterone concentrations were negatively related to PFTriDA and PFTeA levels in adult black-legged kittiwakes and argued that long-term exposure to other long-chain PFCAs (e.g., PFDoA) in some Arctic seabirds could lead to disrupted incubating behavior via reduced estradiol expression.^80^ For Pacific seabirds, data remains limited, but information on biological effects of certain PFAS congeners in rhinoceros auklet embryos sampled from the Pacific coast of BC in 2018 indicate subtle effects on the expression of some biotransformation genes.^33^

## Supporting Information Available

Map of the seabird sampling sites, candidate models and their outputs, as well as additional temporal trend plots, including those for non-detected (i.e., censored) PFASs (PDF)

## Notes

The entire contents of this image have been produced by the main author (Robert Kesic) using a combination of RStudio and PowerPoint. The map, trend plots, and seabird images were all made programmatically in RStudio.
