# Supplementary material for: Perfluoroalkyl Substances in Seabird Eggs from Canada’s Pacific Coast: Temporal Trends (1973–2019) and Interspecific Patterns
Source: Environ Sci Technol. 2023 Jul 13;57(29):10792–803. doi: 10.1021/acs.est.3c02965 (PMC10373494; doi:10.1021/acs.est.3c02965)
Supplement: Supplementary file 1 — es3c02965_si_001.pdf [file es3c02965_si_001.pdf]

Perfluoroalkyl Substances (PFASs) in Seabird Eggs from Canada's Pacific Coast: Temporal Trends (1973–2019) and Interspecific Patterns

Robert Kesic,[a,*] John E. Elliott,[a] Kyle H. Elliott,[b] Sandi L. Lee,[a] France Maisonneuve[c]

[a]Environment and Climate Change Canada, Wildlife Research Division, Delta, British Columbia, Canada: rkesic@sfu.ca (or rkesicc@gmail.com); john.elliott@ec.gc.ca; sandi.lee@ec.gc.ca

[b]Department of Natural Resource Sciences, McGill University, Sainte Anne-de-Bellevue, Quebec, Canada: kyle.elliott@mcgill.ca

[c]Environment and Climate Change Canada, National Wildlife Research Centre, Ottawa, Ontario, Canada: france.maisonneuve@ec.gc.ca

Supporting Information

Pages: 15

This Supporting Information (SI) is 15 pages and includes a map of the seabird sampling sites, 4 tables of all the candidate models and their outputs for each species, as well as extra temporal trend plots for various perfluoroalkyl sulfonates (PFSAs) and perfluoroalkyl carboxylates (PFCAs), including non-detects.

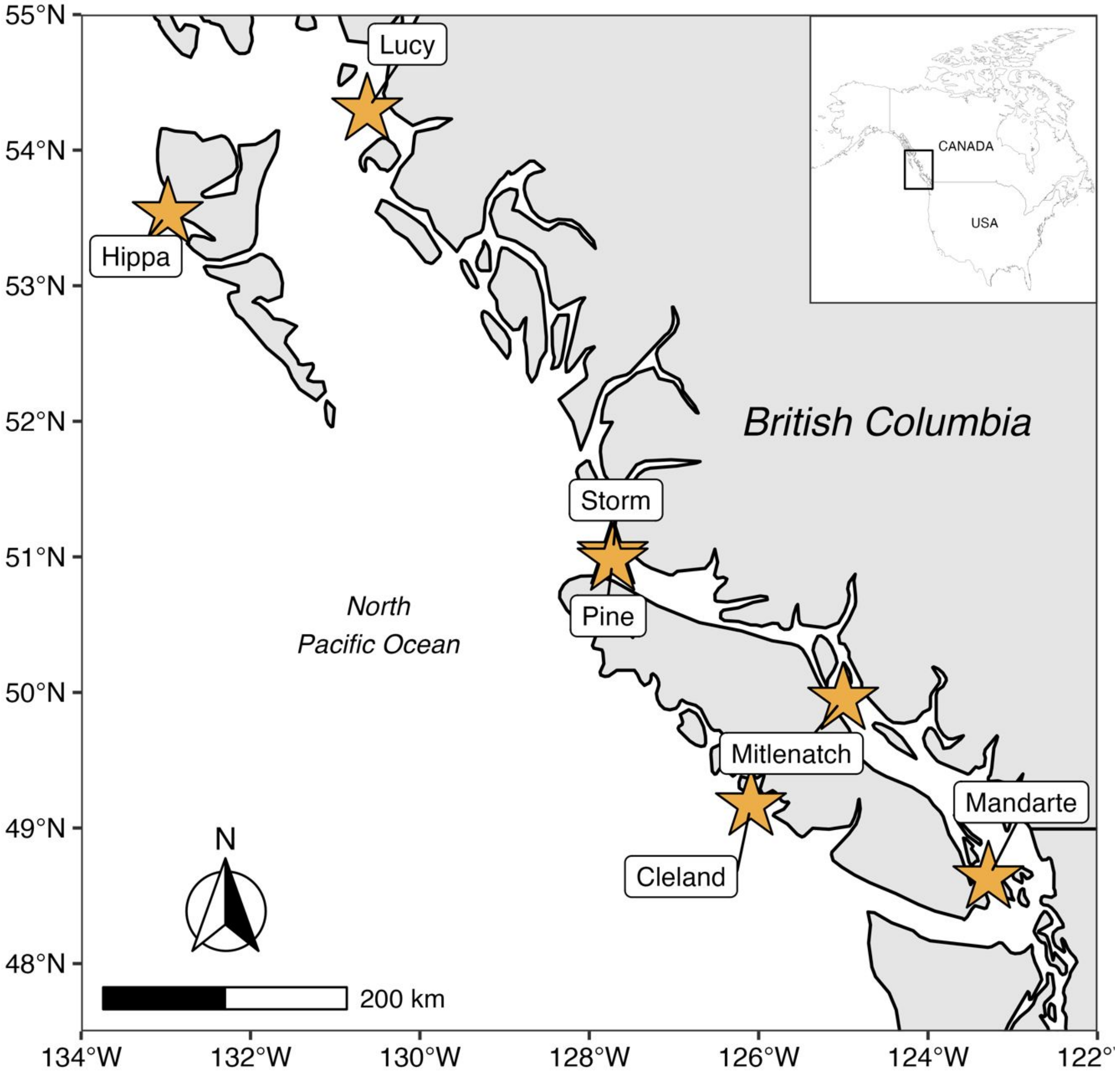

SI Figure 1. Map of seabird colonies and egg collection sites (n = 7) sampled between 1973–2019 from the Pacific coast of British Columbia (BC), Canada. The spatial extent of sampling sites is indicated by the rectangle in the inset (top right corner). Double-crested cormorant colony: Mandarte. Pelagic cormorant colony: Mitlenatch. Rhinoceros auklet colonies: Cleland, Lucy, Pine. Leach's storm-petrel colonies: Cleland, Storm, Hippa.

SI Table 1. Akaike's Information Criterion (AICc, corrected for small sample sizes) values for Generalized Additive Models (GAMs) explaining the variation in fully-detected (i.e. non-censored) PFAS concentrations in the eggs of double-crested cormorants (DCCO) breeding at Mandarte Island in 1973, 1985, 1990, 1994, 1998, 2002, 2006, 2011, 2015, and 2019. Models shown below are representative of the trends in PFAS concentrations with each PFAS/PFAS group (ln-transformed) analyzed separately as the dependent variable with either a non-linear term (smoothing parameter) to describe year and egg $\delta^{13}C$ (carbon) and $\delta^{15}N$ (nitrogen) as independent variables.

**Compound**	**Effective degrees of freedom (edf)**	**Model**	**Log Likelihood**	**AIC**	**ΔAICc**	**Weight**	**R^2**
PFOS	3.53	s(year) + $\delta^{13}C$	-1.66	23.2	0.00	0.766	0.84
PFNA	-	$\delta^{13}C$	-9.2	25.9	0.00	0.145	0.00
PFTriDA	-	$\delta^{13}C$	-11.8	31.0	0.00	0.499	0.20
PFDS	4.14	s(year) + $\delta^{13}C$	-1.17	25.6	0.00	0.505	0.91
Σ_4PFSAs	3.77	s(year) + $\delta^{13}C$	-1.03	23.0	0.00	0.751	0.85
Σ_{13}PFCAs	-	$\delta^{13}C$ + $\delta^{15}N$	-11.6	33.7	0.00	0.446	0.30

SI Table 2. Akaike's Information Criterion (AICc, corrected for small sample sizes) values for Generalized Additive Models (GAMs) explaining the variation in fully-detected (i.e. non-censored) PFAS concentrations in the eggs of pelagic cormorants (PECO) breeding at Mitlenatch Island in 2007, 2011, 2015, and 2019. Models shown below are representative of the trends in PFAS concentrations with each PFAS/PFAS group (ln-transformed) analyzed separately as the dependent variable with either a non-linear term (smoothing parameter) to describe year and egg $\delta^{13}C$ (carbon) and $\delta^{15}N$ (nitrogen) as independent variables.

**Compound**	**Effective degrees of freedom (edf)**	**Model**	**Log Likelihood**	**AIC**	**ΔAICc**	**Weight**	**R^2**
PFOS	2.86	s(year)	-12.6	39.0	0.00	0.507	0.70
	2.79	s(year) + $\delta^{13}C$	-11.1	39.7	0.76	0.347	0.71
PFDA	-	$\delta^{13}C$	-19.6	46.7	0.00	0.499	0.35
	2.84	s(year) + $\delta^{13}C$	-15.3	48.4	1.68	0.215	0.50
PFUdA	2.89	s(year) + $\delta^{13}C$	-6.72	31.4	0.00	0.787	0.77
Σ_4PFSAs	2.86	s(year)	-12.2	38.2	0.00	0.467	0.70
	2.78	s(year) + $\delta^{13}C$	-10.5	38.6	0.38	0.385	0.72
Σ_{13}PFCAs	2.84	s(year) + $\delta^{13}C$	-7.00	31.7	0.00	0.354	0.65
	-	$\delta^{13}C$	-12.2	31.9	0.13	0.332	0.50
	-	$\delta^{13}C$ + $\delta^{15}N$	11.01	32.7	0.95	0.220	0.53

SI Table 3. Akaike's Information Criterion (AICc, corrected for small sample sizes) values for Generalized Additive Models (GAMs) explaining the variation in fully-detected (i.e. non-censored) PFAS concentrations in the eggs of rhinoceros auklets (RHAU) breeding at Cleland, Lucy, and Pine Islands in 1990, 1994, 1995, 1998, 1999, 2002, 2003, 2006, 2007, 2010, 2014, 2018, and 2019. Models shown below are representative of the trends in PFAS concentrations with each PFAS/PFAS group (ln-transformed) analyzed separately as the dependent variable with either a non-linear term (smoothing parameter) to describe year, breeding colony location as a fixed effect, as well as egg $\delta^{13}C$ (carbon) and $\delta^{15}N$ (nitrogen) as independent variables.

**Compound**	**Effective degrees of freedom (edf)**	**Model**	**Log Likelihood**	**AIC**	**ΔAICc**	**Weight**	**R^2**
PFOS	8.64	s(year) + location	13.5	5.8	0.00	0.487	0.90
	8.51	s(year) + location + $\delta^{13}C$	14.9	6.8	0.98	0.299	0.90
PFNA	3.71	s(year) + location + $\delta^{13}C$	0.68	19.4	0.00	0.341	0.89
	4.18	s(year) + $\delta^{13}C$	-1.58	19.7	0.31	0.293	0.88
	4.23	s(year) + $\delta^{13}C$ + $\delta^{15}N$	2.98	20.5	1.12	0.195	0.88
PFDA	5.68	s(year) + location	6.11	11.3	0.00	0.593	0.87
PFUdA	3.22	s(year) + location + $\delta^{13}C$	12.8	-6.1	0.00	0.421	0.86
	3.24	s(year) + location	11.1	-5.5	0.58	0.315	0.85
PFDoA	7.40	s(year) + location	29.3	-29.9	0.00	0.473	0.92
PFTriDA	7.31	s(year) + location	16.8	-5.2	0.00	0.424	0.87
	8.11	s(year) + location + $\delta^{13}C$	19.5	-4.7	0.50	0.330	0.88
	8.47	s(year) + location + $\delta^{15}N$	19.4	-3.3	1.84	0.168	0.88
PFDS	8.91	s(year) + location + $\delta^{13}C$ + $\delta^{15}N$	6.70	27.2	0.00	0.949	0.95
Σ_4PFSAs	8.71	s(year) + location	14.7	3.5	0.00	0.435	0.91
	8.83	s(year) + location + $\delta^{15}N$	16.3	4.1	0.52	0.335	0.91
Σ_{13}PFCAs	3.64	s(year) + location + $\delta^{13}C$	29.9	-39.2	0.00	0.594	0.89
	3.69	s(year) + location + $\delta^{13}C$ + $\delta^{15}N$	30.4	-37.2	1.98	0.221	0.89

SI Table 4. Akaike's Information Criterion (AICc, corrected for small sample sizes) values for Generalized Additive Models (GAMs) explaining the variation in fully-detected (i.e. non-censored) PFAS concentrations in the eggs of Leach's storm-petrels (LSPE) breeding at Cleland, Lucy, and Pine Islands in 1990, 1991, 1994, 1995, 1998, 1999, 2002, 2003, 2006, 2007, 2011, 2015, and 2019. Models shown below are representative of the trends in PFAS concentrations with each PFAS/PFAS group (ln-transformed) analyzed separately as the dependent variable with either a non-linear term (smoothing parameter) to describe year, breeding colony location as a fixed effect, as well as egg $\delta^{13}C$ (carbon) and $\delta^{15}N$ (nitrogen) as independent variables.

**Compound**	**Effective degrees of freedom (edf)**	**Model**	**Log Likelihood**	**AIC**	**ΔAICc**	**Weight**	**R^2**
PFOS	7.88	s(year) + $\delta^{13}C$	-10.5	48.9	0.00	0.251	0.96
	7.39	s(year) + $\delta^{15}N$	-11.4	49.1	0.18	0.229	0.95
	7.52	s(year) + location	-9.56	49.1	0.24	0.223	0.96
	7.72	s(year)	-12.9	50.0	1.16	0.141	0.95
PFNA	4.01	s(year) + location	3.58	11.9	0.00	0.541	0.86
PFUdA	5.31	s(year)	27.5	-37.9	0.00	0.478	0.90
PFTriDA	4.24	s(year)	0.183	14.0	0.00	0.402	0.60
	3.98	s(year) + $\delta^{13}C$	0.531	15.2	1.25	0.215	0.59
Σ_4PFSAs	7.88	s(year) + $\delta^{13}C$	-10.4	48.6	0.00	0.256	0.96
	7.38	s(year) + $\delta^{15}N$	-11.2	48.7	0.12	0.241	0.95
	7.50	s(year) + location	-9.50	48.9	0.32	0.219	0.96
	7.71	s(year)	-12.9	50.0	1.38	0.129	0.95
Σ_{13}PFCAs	5.11	s(year)	23.0	-29.4	0.00	0.469	0.86
	5.21	s(year) + $\delta^{13}C$	23.6	-27.5	1.95	0.177	0.86

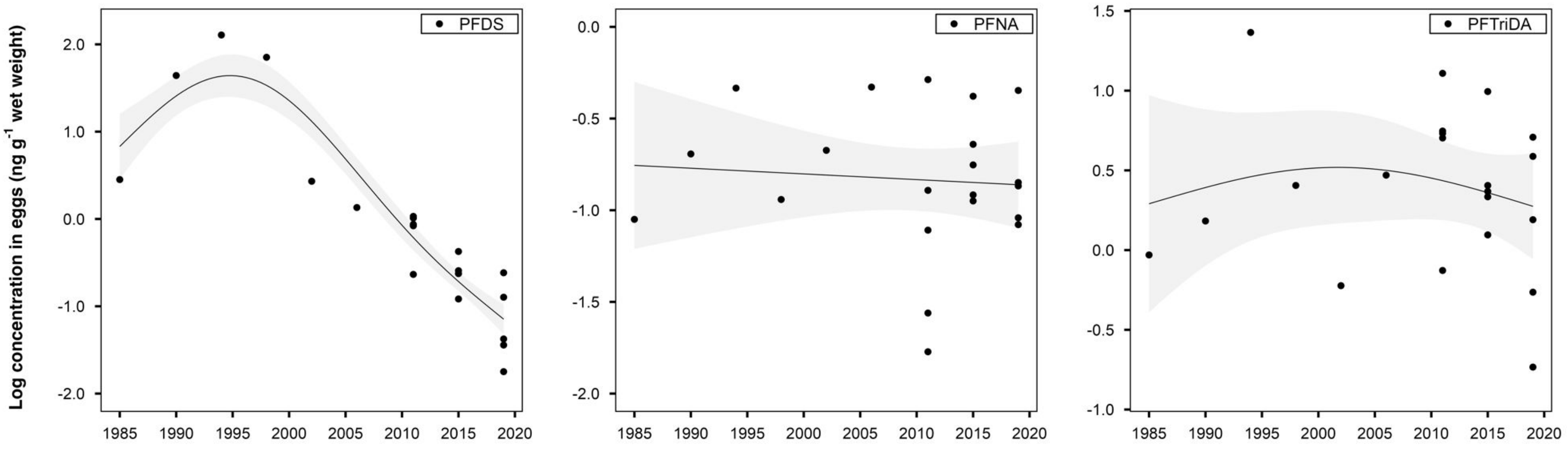

SI Figure 2. Temporal trends for fully-detected (i.e. non-censored) perfluoroalkyl sulfonates (PFSAs) and perfluoroalkyl carboxylates (PFCAs) in the eggs of a nearshore indicator seabird species, the double-crested cormorant (*Nannopterum auritum*; DCCO), sampled between 1985-2019 from Mandarte Island, British Columbia (BC), Canada. Individual dots represent annual concentrations in egg pool samples. Trend lines and 95% prediction intervals (shaded) are fitted using Generalized Additive Models (GAMs).

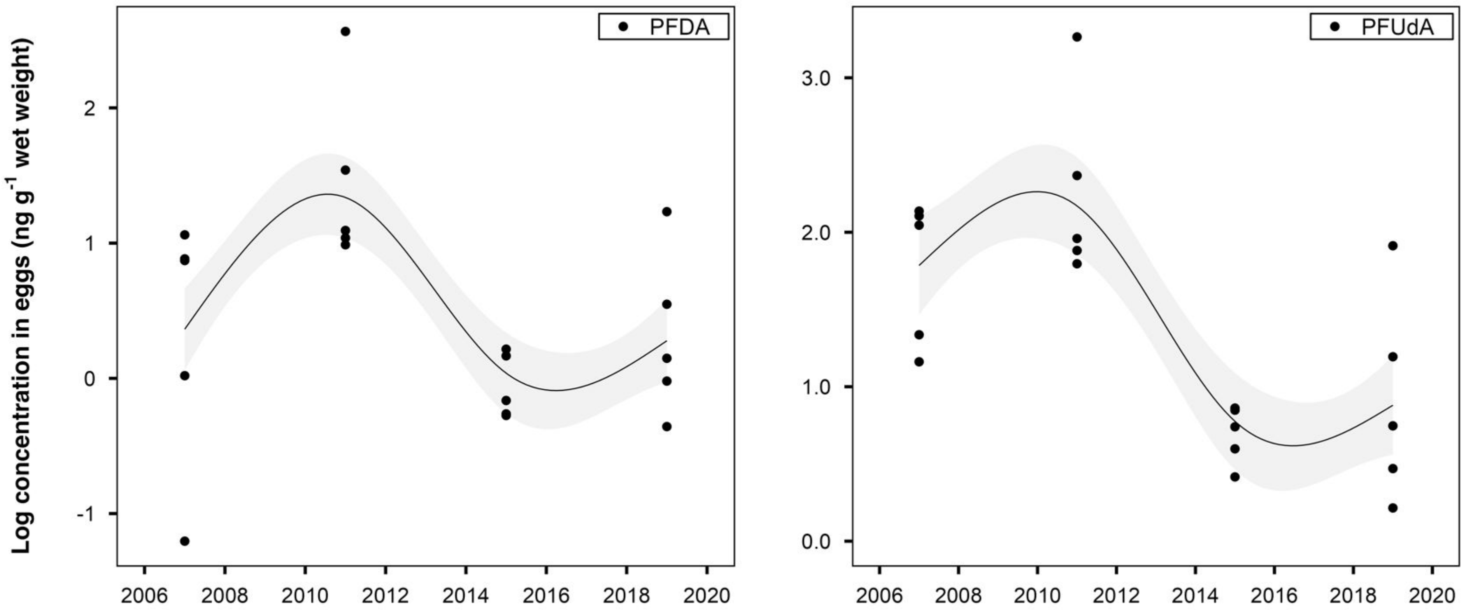

SI Figure 3. Temporal trends for fully-detected (i.e. non-censored) perfluoroalkyl sulfonates (PFSAs) and perfluoroalkyl carboxylates (PFCAs) in the eggs of a nearshore-pelagic indicator seabird species, the pelagic cormorant (*Urile pelagicus*; PECO), sampled between 2007-2019 from Mitlenatch Island, British Columbia (BC), Canada. Individual dots represent annual concentrations in egg pool samples. Trend lines and 95% prediction intervals (shaded) are fitted using Generalized Additive Models (GAMs).

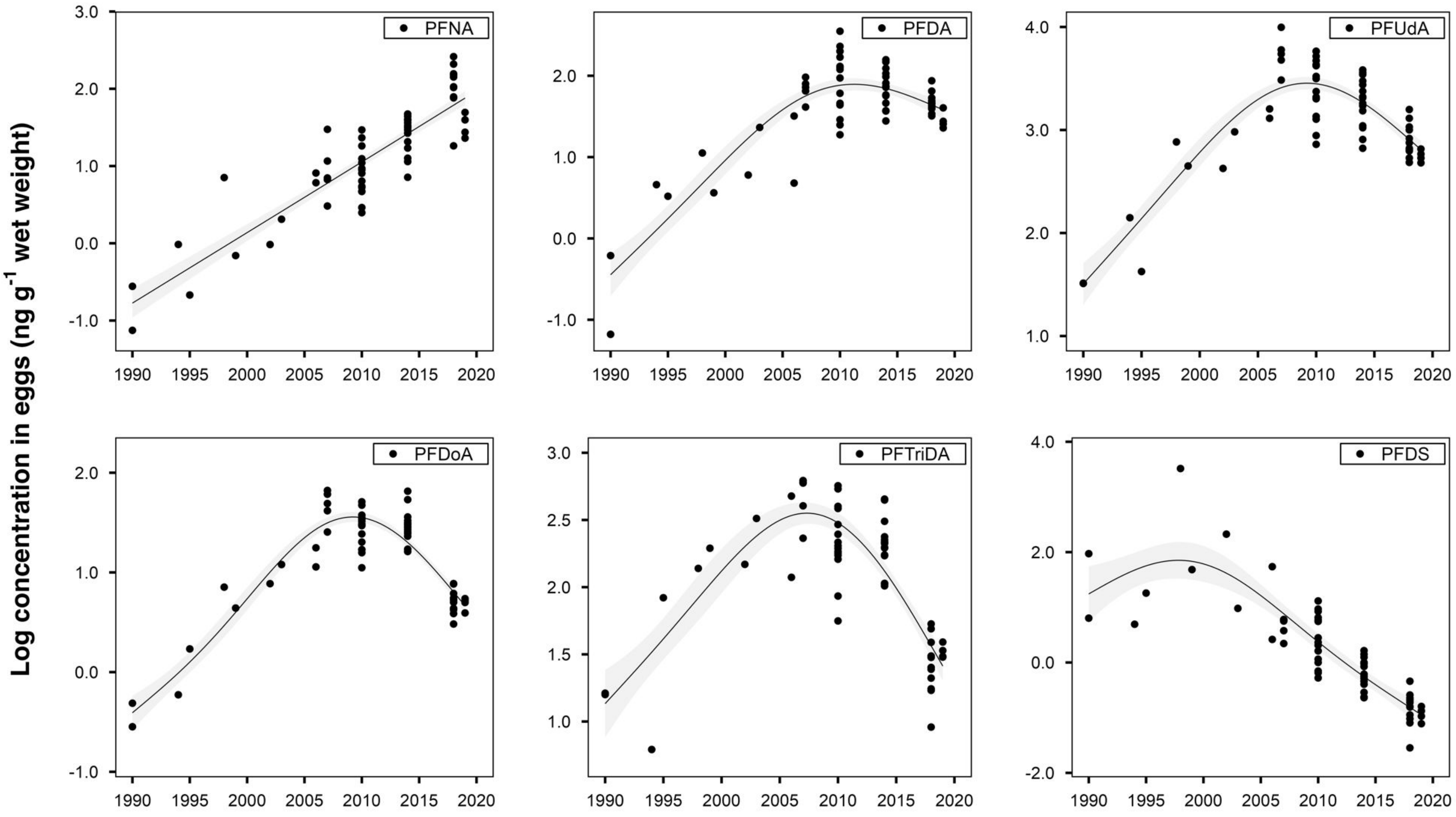

SI Figure 4. Temporal trends for fully-detected (i.e. non-censored) perfluoroalkyl sulfonates (PFSAs) and perfluoroalkyl carboxylates (PFCAs) in the eggs of a continental shelf indicator seabird species, the rhinoceros auklet (*Cerorhinca monocerata*; RHAU), sampled between 1990-2019 from Cleland, Lucy, and Pine Islands from British Columbia (BC), Canada. Individual dots represent annual concentrations in egg pool samples. Trend lines and 95% prediction intervals (shaded) are fitted using Generalized Additive Models (GAMs).

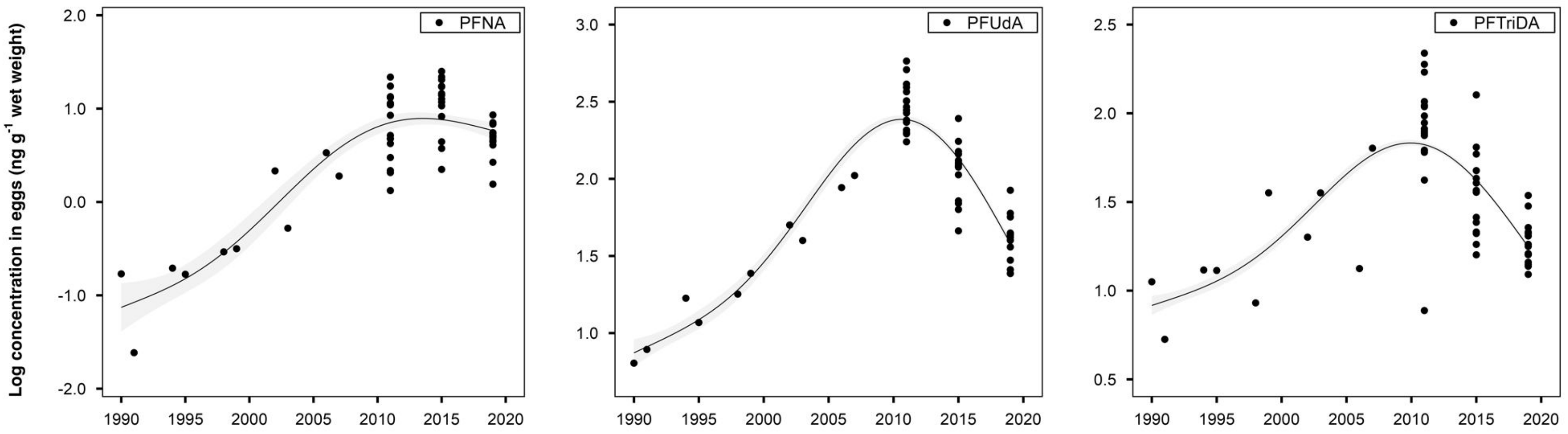

SI Figure 5. Temporal trends for fully-detected (i.e. non-censored) perfluoroalkyl sulfonates (PFSAs) and perfluoroalkyl carboxylates (PFCAs) in the eggs of an offshore/pelagic seabird species, the Leach's storm-petrel (*Hydrobates leucorhous*; LSPE), sampled between 1990-2019 from Cleland, Storm, and Hippa Islands from British Columbia (BC), Canada. Individual dots represent annual concentrations in egg pool samples. Trend lines and 95% prediction intervals (shaded) are fitted using Generalized Additive Models (GAMs).

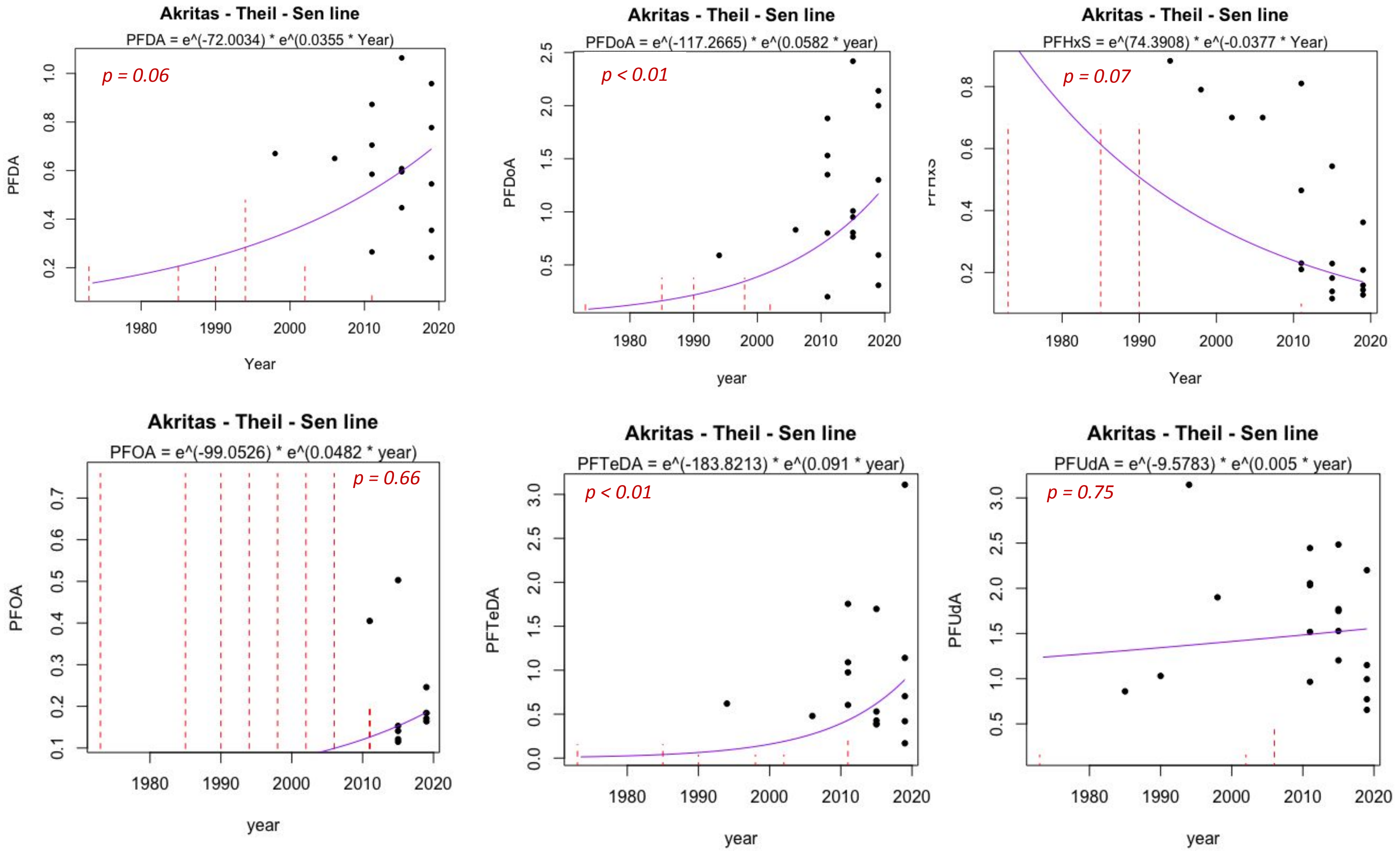

SI Figure 6. Temporal trends for censored (i.e., < Method Detection Limit) PFAS compounds in eggs of double-crested cormorants (DCCO) breeding at Mandarte Island between 1973 and 2019. Black dots represent individual data points. Red dotted lines indicate censored concentration values. Purple solid lines represent the Akritas-Theil-Sen trend line for censored data[1].

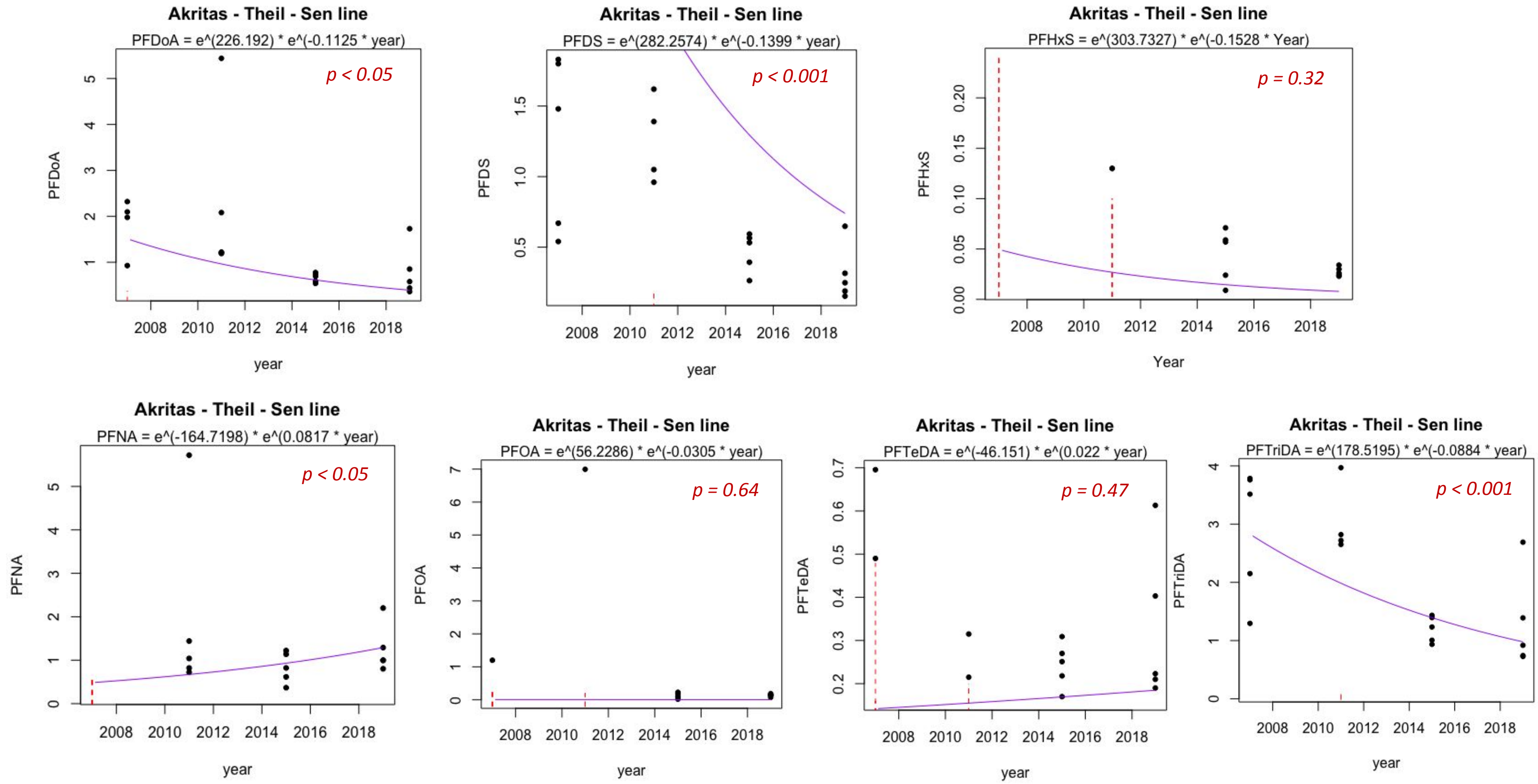

SI Figure 7. Temporal trends for censored (i.e., < Method Detection Limit) PFAS compounds in eggs of pelagic cormorants (PECO) breeding at Mitlenatch Island between 2007 and 2019. Black dots represent individual data points. Red dotted lines indicate censored concentration values. Purple lines represent the Akritas-Theil-Sen trend line for censored data[1].

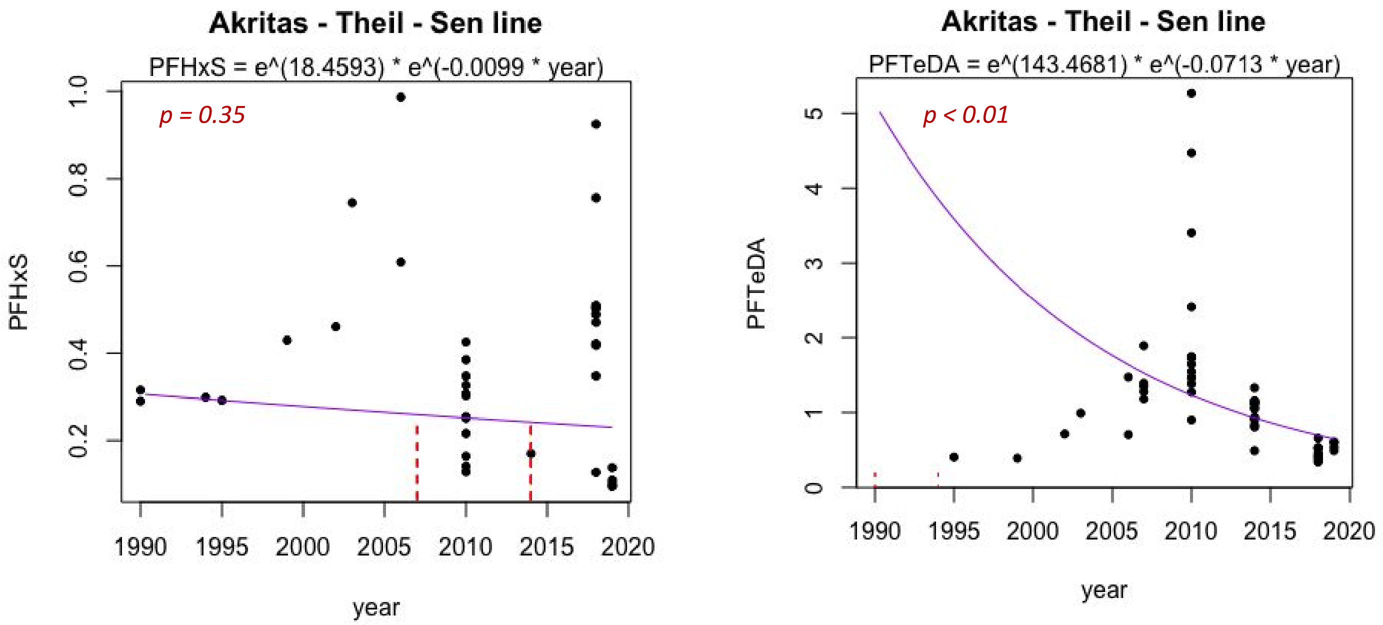

SI Figure 8. Temporal trends for censored (i.e., < Method Detection Limit) PFAS compounds in eggs of rhinoceros auklets (RHAU) breeding at Cleland, Lucy, and Pine Islands between 1990 and 2019. Black dots represent individual data points. Red dotted lines indicate censored concentration values. Purple lines represent the Akritas-Theil-Sen trend line for censored data[1].

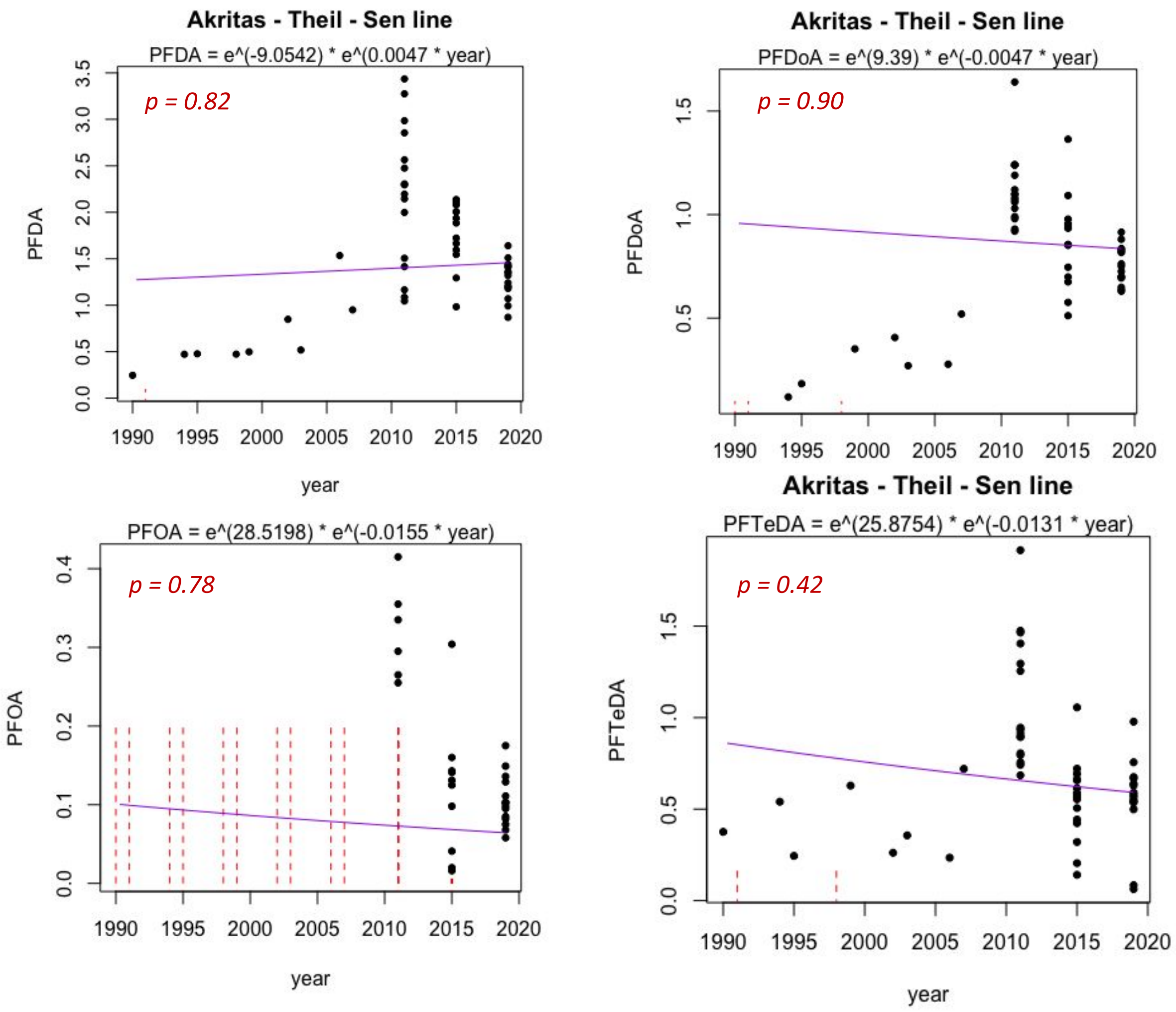

SI Figure 9. Temporal trends for censored (i.e., < Method Detection Limit) PFAS compounds in eggs of Leach's storm-petrels (LSPE) breeding at Cleland, Hippa, and Storm Islands between 1990 and 2019. Black dots represent individual data points. Red dotted lines indicate censored concentration values. Purple lines represent the Akritas-Theil-Sen trend line for censored data[1].

References

1. Julian, P., Helsel, D.R., 2021. NADA2: Data Analysis for Censored Environmental Data.
